# Methanol Metabolism in the Cytochrome and Menaquinone‐Containing Acetogen *Moorella thermoacetica*

**DOI:** 10.1111/1462-2920.70377

**Published:** 2026-07-22

**Authors:** Florian P. Rosenbaum, Anja Poehlein, Rolf Daniel, Volker Müller

**Affiliations:** ^1^ Department of Molecular Microbiology & Bioenergetics, Institute of Molecular Biosciences Johann Wolfgang Goethe University Frankfurt am Main Germany; ^2^ Genomic and Applied Microbiology & Göttingen Genomics Laboratory Georg‐August University Göttingen Göttingen Germany

**Keywords:** acetogenesis, bioenergetics, C1 metabolism, DMSO respiration, energy conservation, energy‐converting hydrogenase, hydrogen, NADH dehydrogenase

## Abstract

Methanol derived from pectin degradation is common in oxic and anoxic sediments and is used by various aerobic and anaerobic microorganisms such as acetogenic bacteria or methanogenic archaea as a carbon and energy source. Acetogens use the Wood‐Ljungdahl pathway to disproportionate methanol to CO_2_ and acetate. Methanol metabolism has been studied primarily in mesophilic acetogens such as *Acetobacterium woodii* and *Eubacterium callanderi,* that do not have cytochromes or quinones. *Moorella thermoacetica* , the model organism for elucidation of acetogenesis, is a thermophilic, cytochrome‐ and quinone‐containing acetogen which is commonly found in terrestrial soil. Here, we elucidated the physiology and bioenergetics of *M. thermoacetica* growing on methanol by performing physiological and biochemical experiments as well as genome‐wide transcription analysis. Thereby, we identified genes and enzymes involved in acetogenesis from methanol. We will present a comprehensive model for carbon and energy flow from methanol to acetate and line out the bioenergetics of methanol‐based acetogenesis in *M. thermoacetica* . Furthermore, we show that in the presence of DMSO as an alternative electron sink, no acetate is produced from methanol.

## Graphical abstract

The thermophilic acetogenic bacterium *Moorella thermoacetica* is a promising platform organism to convert C1 compounds to valuable products. Gaseous C1 substrates are technically difficult to use for fermentation in an industrial scale, but methanol, which can be produced chemically from carbon dioxide, is a suitable alternative. However, little is known about methanol metabolism and its bioenergetics in *M. thermoacetica* . To fill this gap, we performed physiological studies, genome‐wide expression analyses and enzymatic assays.

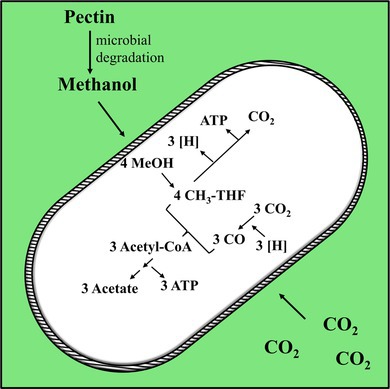

## 1 Introduction

Acetogenic bacteria are a phylogenetically diverse group of strict anaerobs that use C1 compounds such as CO_2_, CO, formate or methyl groups to produce acetate (Drake et al. 2008). The main carbon pathway in these bacteria is the Wood‐Ljungdahl pathway (WLP) (Wood et al. 1986). Briefly, the WLP consists of two separate branches: the methyl branch and the carbonyl branch and in each branch, one molecule of carbon dioxide is reduced (Ljungdahl 1986; Wood et al. 1986). While the carbonyl branch comprises only a single enzymatic reaction, the reduction of carbon dioxide to enzyme‐bound carbon monoxide by the CO dehydrogenase/acetyl‐CoA synthase (CO‐DH/ACS), the methyl branch involves several steps (Ljungdahl 1986; Wood et al. 1986). The initial reaction is the reduction of CO_2_ to formate by a formate dehydrogenase/CO_2_ reductase. This reaction poses an energetic hurdle because the redox potential of the CO_2_/formate couple is rather low (*E*_0_′ = −432 mV) (Thauer et al. 1977) and NAD^+^/NADH (*E*_0_′ = −320 mV; *E*' = −270 mV) (Thauer et al. 1977) cannot drive CO_2_ reduction. In *Moorella thermoacetica* , however, CO_2_ reduction to formate is catalysed by a NADPH‐dependent formate dehydrogenase (Yamamoto et al. 1983). The intracellular redox potential of the NADP^+^/NADPH couple (*E*' = −370 mV) (Huang et al. 2012) is similar to that of the CO_2_/formate couple. Next, formate is bound to the C1 carrier tetrahydrofolate (THF) at the expense of one ATP (Sun et al. 1969; Ljungdahl et al. 1970). Formyl‐THF is then further metabolised to methenyl‐THF and further reduced to methylene‐THF (O'Brien et al. 1973; Clark and Ljungdahl 1982). The reduction of methylene‐THF to methyl‐THF is catalysed by the methylene‐THF reductase (MTHFR) and currently four different MTHFRs with very different subunit compositions are known in acetogenic bacteria (Öppinger et al. 2022). *M. thermoacetica* has a type IV‐enzyme that is suggested to use the mode of electron bifurcation/confurcation with NADH and methylene‐THF and a third yet unknown electron carrier (Mock et al. 2014). It is hypothesised that a membrane‐bound electron carrier such as a cytochrome or quinone may be the third electron carrier (Mock et al. 2014; Kremp et al. 2022; Rosenbaum et al. 2026). Finally, the methyl group is bound to a corrinoid iron–sulfur protein (CoFeSP) (Drake et al. 1981). The key enzyme of the pathway, the CO‐DH/ACS, catalyses the condensation of methyl‐CoFeSP and enzyme‐bound CO as well as coenzyme A (CoA), resulting in acetyl‐CoA (Ragsdale et al. 1982, 1983; Ragsdale and Wood 1985). Acetyl‐CoA can be used either for biomass production or is further metabolised to acetyl phosphate and acetate, which is coupled to ATP synthesis (Wood et al. 1986). The ATP balance of acetogenesis by substrate level phosphorylation is zero; additional ATP is gained by a chemiosmotic mechanism (Schuchmann and Müller 2014). The electrochemical ion gradient is established by two different respiratory enzyme complexes, either the Rnf or Ech complex (Müller 2003). Both are fueled by reduced ferredoxin, with the Rnf complex using NAD^+^ and the Ech complex using protons as electron acceptor (Biegel and Müller 2010; Schoelmerich and Müller 2019; Katsyv and Müller 2022). Electron transport is coupled to drive the export of ions (Na^+^, H^+^), thus creating an electrochemical ion potential across the membrane that drives ATP synthesis (Reidlinger and Müller 1994; Biegel and Müller 2010; Schoelmerich and Müller 2019; Litty and Müller 2022). In the reverse mode, the electrochemical ion gradient established by ATP hydrolysis drives the Rnf‐ or Ech complex to reduce ferredoxin using electrons derived from NADH or H_2_, respectively (Bertsch et al. 2016; Kremp et al. 2018; Westphal et al. 2018).

**FIGURE 1:**
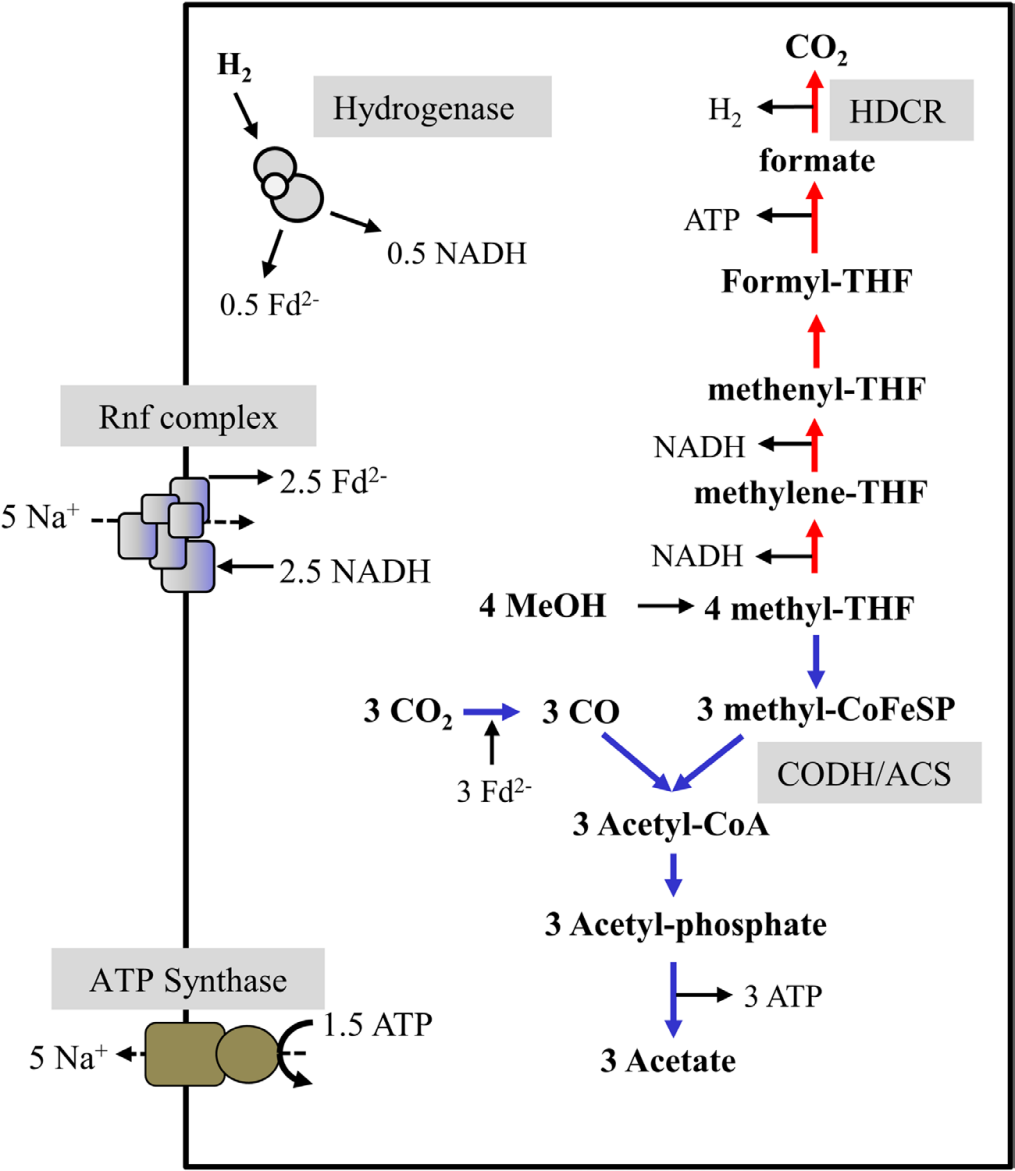
Biochemistry and bioenergetics of acetogenesis from methanol + CO_2_ in *A. woodii* . A stoichiometry of 2 H^+^/2 e^−^ is assumed for the Rnf complex. The stoichiometry of the ATP synthase is 3.3 Na^+^/ATP (Matthies et al. 2014). Fd, ferredoxin; THF, tetrahydrofolate; CODH/ACS, CO dehydrogenase/acetyl coenzyme A synthase; HDCR, hydrogen‐dependent CO_2_ reductase.

Formate and methanol are often regarded as liquid CO_2_ equivalents and are of great use for biotechnological applications since the problems inherent to gas fermentation are circumvented. Methanol metabolism in thermophilic acetogens has received only little attention, but was studied in detail in the mesophilic, Rnf‐containing bacteria *Eubacterium callanderi* and *Acetobacterium woodii* (Kremp et al. 2018; Keller et al. 2019; Dietrich et al. 2021; Kremp and Müller 2021; Litty et al. 2022; Bae et al. 2025; Vecchini Santaella et al. 2025). In general, 4 mol of methanol are converted to 4 mol of methyl‐THF by a methanol‐specific methyltransferase system. One mol of methyl‐THF is oxidised to carbon dioxide, releasing six electrons (Figure 1). These six electrons are used to reduce 3 mol of carbon dioxide to enzyme‐bound carbon monoxide. From the remaining 3 mol of methyl‐THF the methyl group is transferred to CoFeSP and then condensed with enzyme‐bound CO by the CO‐DH/ACS to acetyl‐CoA. The resulting acetyl‐CoA is further metabolised to 3 mol acetate, yielding 3 mol of ATP. In total, 4 mol methanol are converted to 3 mol acetate and 2.5 mol ATP, giving an ATP:acetate ratio of 0.8 or a ATP:methanol ratio of 0.63.

*M. thermoacetica* is a thermophilic, cytochrome‐ and quinone‐containing acetogen (Rosenbaum and Müller 2023). It does not use a Rnf complex as a respiratory enzyme, but rather uses a putative Ech‐formate dehydrogenase complex (Fdh‐Ech) and/or a ‘headless’ ferredoxin‐dependent NADH dehydrogenase (Rosenbaum et al. 2026). Furthermore, methylene‐THF is reduced in *M. thermoacetica* by a putative electron‐bifurcating MTHFR that uses NADH and a second yet unknown electron donor (Mock et al. 2014; Rosenbaum et al. 2026). Additionally, CO_2_ is not reduced to formate in *M. thermoacetica* by the hydrogen‐dependent carbon dioxide reductase like in *A. woodii*; instead, a NADP^+^‐dependent formate dehydrogenase is used (Yamamoto et al. 1983; Schuchmann et al. 2016). Interestingly, the genome of *M. thermoacetica* encodes two soluble hydrogenases, the electron‐bifurcating hydrogenase and a NADP^+^‐reducing hydrogenase (Pierce et al. 2008; Rosenbaum and Müller 2024). In contrast to *A. woodii* , *M. thermoacetica* can conserve extra energy by reducing DMSO using a membrane‐bound DMSO reductase (Rosenbaum et al. 2022). As the enzymes and electron carriers involved in the WLP in *M. thermoacetica* and *A. woodii* differ considerably, one could assume a different ATP yield in *M. thermoacetica* compared to acetogens that do not have cytochromes or quinones. To unravel the bioenergetics of acetogenesis from methanol, we have analysed the biochemistry and bioenergetics of this process in *M. thermoacetica* . We will present a coherent metabolic scheme for methanol metabolism in this thermophilic acetogenic model bacterium.

## 2 Results

### 2.1 Growth of *M. thermoacetica* on Methanol

**FIGURE 2:**
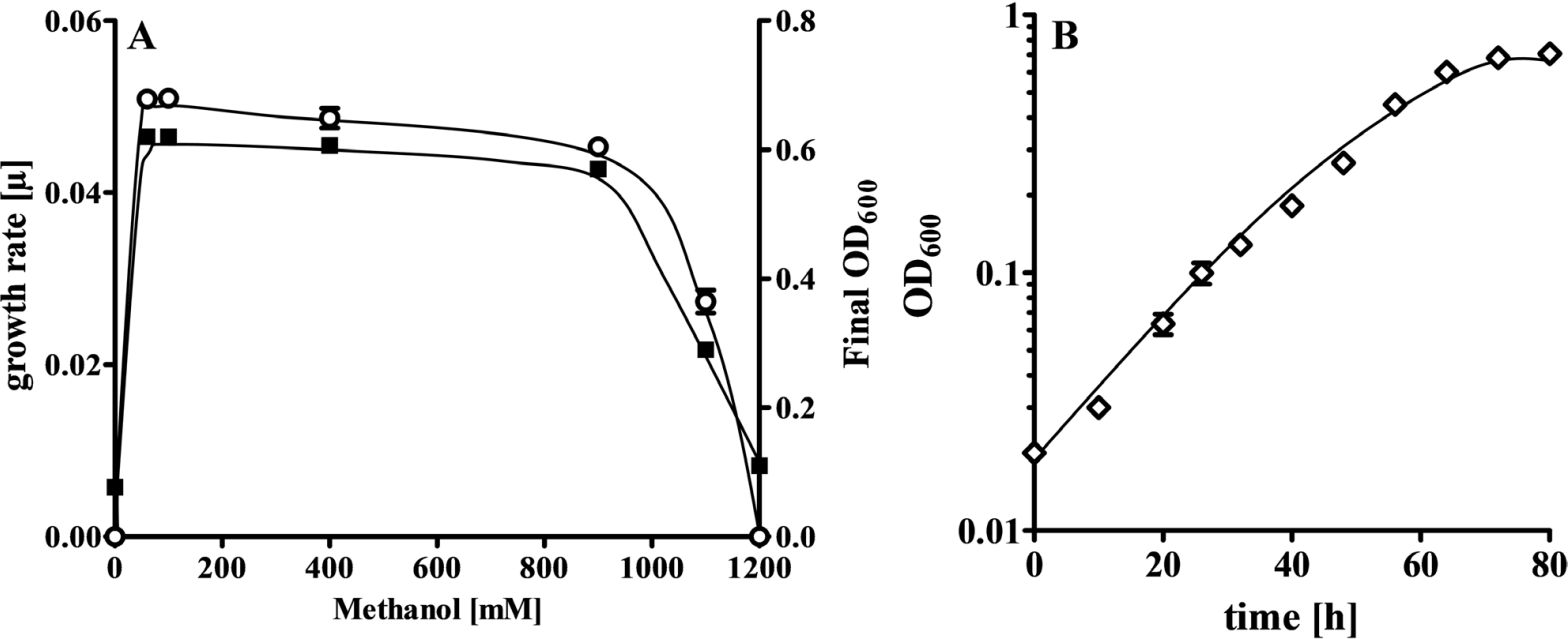
Growth of *M. thermoacetica* on methanol. Cells were cultivated in 5 mL bicarbonate‐buffered medium under a N_2_ + CO_2_ (80:20 [v/v]) atmosphere. (A) Effect of increasing methanol concentration on growth of *M. thermoacetica* . The growth rate (○) and final OD_600_ (■) are plotted against the methanol concentration. (B) Growth of *M. thermoacetica* on methanol. Cells were cultivated with 60 mM methanol and the OD_600_ (◊) was monitored over time (*n* = 3, SD).

First, cells of *M. thermoacetica* were adapted to use methanol as the sole carbon and energy source through three consecutive transfers in media containing 60 mM methanol as sole carbon and energy source. The final OD and growth rate were dependent on the methanol concentration, reaching a maximum at 60 mM. Up to 900 mM methanol, the growth rate and final OD decreased only slightly; at 1.1 M, both parameters were reduced by approximately 50%. There was no growth at 1.2 M methanol (Figure 2A). All further growth experiments were done with 60 mM methanol; a representative growth curve is given in Figure 2B, the growth rate (μ) was 0.05 h^−1^ (doubling time = 13.6 h) and the final OD 0.62 **±** 0.03.

### 2.2 DMSO Is the Preferred Electron Sink During Methanol Oxidation

Unlike many acetogens, *M. thermoacetica* can reduce terminal electron acceptors other than CO_2_, such as DMSO, using glucose or H_2_ as an electron donor (Rosenbaum et al. 2022; Rosenbaum and Müller 2024). Thermodynamically, DMSO respiration is much more favourable because the redox couple DMS/DMSO (*E*_0_′ = +160 mV) has a much more positive redox potential than, for example, formate/CO_2_ (*E*_0_′ = −432 mV) (Thauer et al. 1977). Reduction of DMSO as an alternative electron acceptor therefore enables additional energy conservation. This is of particular interest for a C1‐based bioeconomy, as the major bottleneck associated with the conversion of C1 substrates is their inherently low energy yield for the cells.

**FIGURE 3:**
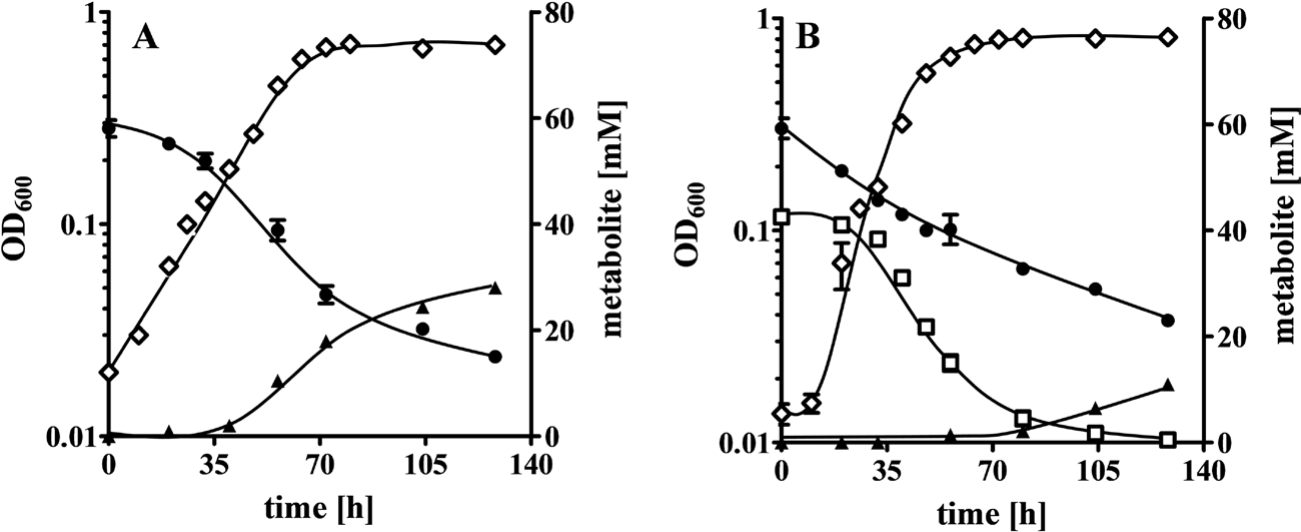
DMSO reduces acetate formation by *M. thermoacetica* during methanol metabolism. Cells were grown in bicarbonate‐buffered medium under a N_2_ + CO_2_ (80:20 [v/v]) atmosphere at 55°C with 60 mM methanol in the absence (A) or presence (B) of 40 mM DMSO. OD_600_ (◊), methanol (●), acetate (▲) and DMSO (□) concentrations were monitored (*n* = 3; SD).

Therefore, we addressed the question whether methanol oxidation can be linked to DMSO reduction as well and what kind of effect DMSO has on methanol metabolism. Indeed, *M. thermoacetica* reduced DMSO with methanol as electron donor. In the presence of DMSO, the growth rate increased by 56% but the final OD remained nearly the same for cultures grown in the presence or absence of DMSO (Figure 3A,B). Methanol was consumed under both conditions, with acetate as the main product; additionally, traces of hydrogen were detected under both conditions. Less acetate was produced in the presence of DMSO. To our surprise, DMSO was reduced first and only when DMSO was nearly completely consumed, acetate formation started (Figure 3B). Obviously, DMSO was the preferred electron acceptor for methanol oxidation. Thus, we hypothesised that growth should be possible by reduction of DMSO in the absence of CO_2_/bicarbonate and indeed, this was observed (Figure S1).

### 2.3 Methanol Utilisation by Resting Cells of *M. thermoacetica*

**FIGURE 4:**
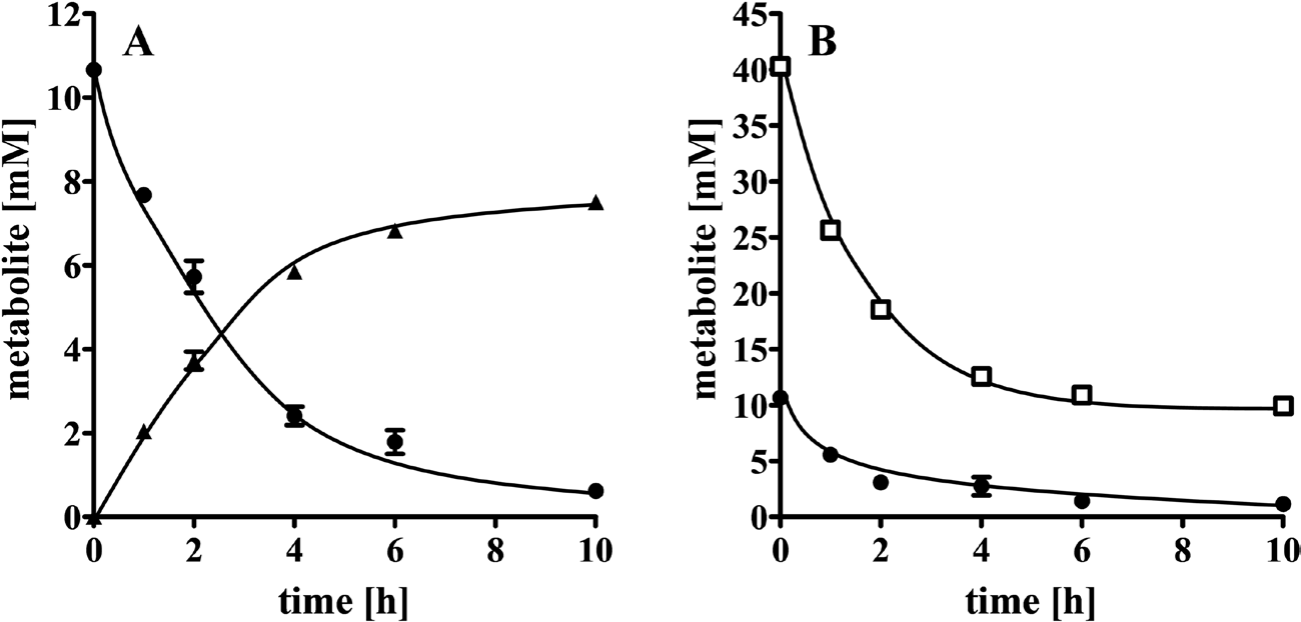
Methanol utilization by resting cells of *M. thermoacetica* in the presence or absence of DMSO. Cells were grown on methanol in the presence or absence of DMSO, harvested in the late exponential growth phase and resting cells were prepared. Cell suspensions were supplemented with (A) 10 mM methanol or (B) 10 mM methanol +40 mM DMSO. The assays were performed in 120 mL serum bottles filled with bicarbonate‐containing resting cell buffer (50 mM MOPS (pH 7.0), 50 mM KHCO_3_, 20 mM NaCl, 20 mM MgSO_4_ × 7 H_2_O, 20 mM KCl, 2 mM DTE, 4 μM resazurin) under a N_2_ + CO_2_ (80:20 [v/v]) gas atmosphere at ambient pressure. The final assay volume was 20 mL and the assays were preincubated at 55°C for 15 min before the reaction was started by the addition of methanol. Methanol (●), acetate (▲) and DMSO (□) concentrations were monitored by GC and HPLC (*n* = 3; SD).

To elucidate the stoichiometry of methanol consumption and acetate production, experiments with resting cells were conducted. In the presence of CO_2_/bicarbonate, resting cells consumed methanol with a rate of 2.98 ± 0.17 mM/h^−1^ and produced acetate with a rate of 2.05 **±** 0.05 mM/h^−1^. Traces of hydrogen (0.08 ± 0.01 mM) were formed; other metabolites such as lactate were not detected. The ratio of methanol consumed to acetate produced was 4:3 (Figure 4A). In the presence of DMSO, methanol consumption was much faster with a rate of 5.10 **±** 0.21 mM/h^−1^. At the same time, DMSO was reduced with a rate of 14.65 **±** 0.52 mM/h^−1^. At the end of the experiment, 9.7 mM of methanol and 30.2 mM DMSO had been consumed, corresponding to a DMSO/methanol ratio of 3, as would be expected if methanol was oxidized to CO_2_. Acetate was not produced by resting cells in the presence of DMSO (Figure 4B).

### 2.4 Identification of Genes Involved in Methanol Metabolism by Transcriptome Analyses

To identify genes involved in methanol metabolism, genome‐wide expression profiling was performed. A log2fold change (log2FC) threshold of +2/−2 and a p‐adjust value threshold of < 0.05 were used to identify a total of 786 differentially expressed genes out of all 2594 in cells grown on methanol compared to glucose (Tables S1 and S2).

Methanol conversion requires the action of a methyltransferase system built up by the substrate‐specific methyltransferase I (MTI, MtaB), a corrinoid protein (CoP, MtaC) and a methyltransferase II (MTII, MtaA) that transfers the methyl group from methyl‐CoP to THF. Typically, these genes form an operon on the chromosome. *M. thermoacetica* has different methyltransferase systems encoding genes and transcript counts of Mothe_c11840 to 11860 were highly increased with values of a log2FC of +9.0 to +9.9, suggesting that MtaA1B1C1 is the methanol‐specific methyltransferase system of *M. thermoacetica* . This is in contrast with a previous report in which MtaA4 was suggested as MTII of the methanol‐specific methyltransferase system of a different *M. thermoacetica* strain ( *M. thermoacetica* ATCC 39073) (Das et al. 2007). Surprisingly, the transcript level of *mtaA4* was drastically reduced with a log2FC of −8.1.

Furthermore, the transcript levels of other methyltransferases‐coding genes involved in, e.g., demethylation of *O*‐methyl compounds such as vanillate were increased as well but to a lower extend. Since methyltransferases use vitamin B_12_ as cofactor, it was not surprising that transcript levels of genes coding for proteins responsible for the vitamin B_12_ biosynthesis were increased (log2FC +2.1 to +3.3). These genes were found in a one single cluster (Mothe_c10470 to 10620). Additionally, the transcript levels of genes encoding a putative Co^2+^ transporter (Mothe_c11970 to 12010) were increased by log2FC of +1.5 to +1.8.

Next, we focused on genes encoding enzymes involved in the WLP. The transcript levels of the methyltransferase AcsE (Mothe_c11730, log2FC = +2.7), MTHFR (Mothe_c11670 to 11720, log2FC = +1.9 to +2.4) and CO‐DH/ACS (Mothe_c11780 to 11790, log2FC = +1.4 to +2.0) were increased and that of the phosphotransacetylase 2 (Mothe_c11560, log2FC = −4.5) was reduced. The transcript levels of other genes encoding WLP enzymes were not altered.

We continued by focusing on genes that encode enzymes involved in redox carrier balancing and energy conservation. The *M. thermoacetica* genome encodes two soluble hydrogenases, an electron‐bifurcating and a NADP^+^‐reducing hydrogenase. Transcript levels of genes encoding the electron‐bifurcating hydrogenase were strongly reduced (Mothe_c17260 to 17280, log2FC = −5.1 to −4.9), while those for the NADP^+^‐reducing hydrogenase were strongly increased (Mothe_c19180 to 19230, log2FC +7.3 to +14.5). Since the latter could not, in vitro, produce H_2_ from NADPH (see below), the observed hydrogen production in the growth‐ or resting‐cell experiments may have been catalysed by the Ech complex. The *ech* genes (Mothe_c22430 to 22510) are preceded by genes encoding a formate dehydrogenase and formate transporter (Mothe_c22520 to 22540) and it is assumed that the Fdh and the Ech form a functional complex (Rosenbaum et al. 2026). However, only transcript levels of the formate dehydrogenase (Mothe_c22520 and 22530, log2FC = +2.7 and +2.1) and the formate transporter (Mothe_c22540, log2FC = +1.4) were increased during growth on methanol. Transcript levels of the genes encoding the second putative respiratory enzyme of *M. thermoacetica* , the Fd‐dependent NADH dehydrogenase, were similar in cells grown on either methanol or glucose. Furthermore, the transcript levels of genes encoding the EtfABCX complex (Mothe_c00620 to 00650, log2FC = −2.5 to −2.2) and the monofunctional CO‐DH (Mothe_c20130, log2FC = −6.5) were reduced. Similarly, transcript levels of genes encoding the Nfn transhydrogenase (Mothe_c15090 to 15100) and the ATP synthase (Mothe_c24560 to 24630) remained consistent in both growth conditions.

Among the genes with a decreased transcript level were those encoding proteins involved in glycolysis/gluconeogenesis, such as the glyceraldehyde‐3‐phosphate dehydrogenase, phosphoglycerate kinase, triosephosphate isomerase, 2,3‐bisphosphoglycerate‐independent phosphoglycerate mutase and enolase (Mothe_c02780 to 02820, log2FC = −2.0 to −3.3). This is expected since glycolytic enzymes are required for growth on glucose. On the other hand, the transcript level of the gene encoding the fructose‐1,6‐bisphosphatase (Mothe_c23350, log2FC = +11.6) was among the most highly increased during methanol metabolism, which is unsurprising given the key role of fructose‐1,6‐bisphosphatase in synthesizing complex sugar, a function required when cells grow on a C1 substrate such as methanol. Furthermore, the transcript level of *ldh*, which encodes the recently purified and characterised NAD^+^‐dependent lactate dehydrogenase (Rosenbaum and Müller 2025) was reduced (Mothe_c18540, log2FC = −5.8). In case of a high carbon and electron pressure, this LDH acts as a carbon and electron shunt by reducing pyruvate with NADH to lactate. The reduced transcript count is not surprising since *M. thermoacetica* requires complex sugars for anabolism, so lactate production is not beneficial.

The transcript levels of several genes encoding transporters for divalent ions were reduced, including a putative Fe^2+^ transporter (Mothe_c14220 to 14240, log2FC = −7.2 to −6.5), Mn^2+^ transporter (Mothe_c14330 to 14350, log2FC = −2.8 to −2.0) and a Ni^2+^ transporter (Mothe_c18930 to 18950, log2FC = −2.3 to −2.0). These ions are required by several enzymes for their catalytic centres, such as [FeFe]‐ or [NiFe]‐hydrogenases or, in general, for electron transport by FeS centre. Reduced transcript levels may indicate that the overall demand for these cations during growth on methanol is lower than during growth on glucose. In contrast, the transcript levels of genes encoding a putative K^+^ transporter (Mothe_13310 to 13340, log2FC +2.3 to +2.0) were increased.

### 2.5 Identification of Genes Involved in DMSO Reduction by *M. thermoacetica* During Methanol Metabolism

Genome‐wide expression profiling was also performed to identify genes involved in DMSO reduction driven by methanol oxidation. A log2fold change (log2FC) threshold of +2/−2 and a p‐adjust value threshold of < 0.05 were used to identify a total of 268 differentially expressed genes out of all 2594 in cells grown on methanol + DMSO compared to methanol alone (Tables S3 and S4).

In the presence of DMSO, the transcript levels of the methanol‐specific methyltransferase system (Mothe_11840 to 11860) remained unchanged. Transcript levels of genes encoding enzymes of the WLP, such as the formyl‐THF synthetase (Mothe_c01150, log2FC = −2.5), MTHFR (Mothe_c11670 to 11720, log2FC = −2.2 to −1.8), methyltransferase AcsE (Mothe_11730, log2FC = −2.6), NADP^+^‐dependent formate dehydrogenase (Mothe_c23870 to 23,890, log2FC = −1.8 to −1.7) and CO‐DH/ACS (Mothe_c11780 to 11,790, log2FC = −2.4) were reduced. This is consistent with the finding that acetate was not produced in the presence of DMSO. Genes coding for the Fdh‐Ech complex, NADH dehydrogenase, ATP synthase, NADP^+^‐reducing hydrogenase and Nfn transhydrogenase were not differentially expressed in the presence of DMSO. On the other hand, transcript levels of genes encoding the EtfABCX complex (Mothe_c00620 to 00650, log2FC +3.7 to +4.3) and the electron‐bifurcating hydrogenase (Mothe_c17260 to 17,280, log2FC +2.5 to +2.7) were increased.

*M. thermoacetica* has three potential DMSO reductase gene clusters (*dmsBA*) that are physically linked (Rosenbaum et al. 2022). Of these three, transcript levels for two gene clusters (*dmsB2A2*, Mothe_c13700 to 13,710, log2FC = +3.3 to +3.4; *dmsB3A3*, Mothe_c13730 to 13,740, +4.2 to +4.5) were increased; the other was not affected. This is consistent with expression data from cells grown on glucose in the presence and absence of DMSO (Rosenbaum et al. 2022).

In addition to the genes with a reduced transcript count that were already discussed, only a handful of further genes with reduced transcript counts were detected during growth on methanol + DMSO. The gene transcript counts of the already mentioned Fe^2+^ transporter encoded by Mothe_c14220 to 14,240 were further reduced by a log2FC of −3.6 to −2.0. Furthermore, the transcript levels of genes encoding a putative glycolate oxidoreductase (Mothe_c23800 to 23,830, log2FC = −2.4 to −2.0) were reduced. Since *M. thermoacetica* can metabolise glycolate as a carbon and energy source, the glycolate oxidoreductase might be involved (Seifritz et al. 1999).

### 2.6 Genetic Organisation and Function of Deduced Proteins of the Methanol‐Dependent Methyltransferase System From *M. thermoacetica*

As mentioned before, substrate‐specific methyltransferase systems require three components that are mostly found as three different polypeptides, MTI, CoP and MTII, encoded by *mtaB*, *mtaC* and *mtaA*. MTI is substrate specific, but CoP and MTII can be shared by different MTI enzymes.

**FIGURE 5:**
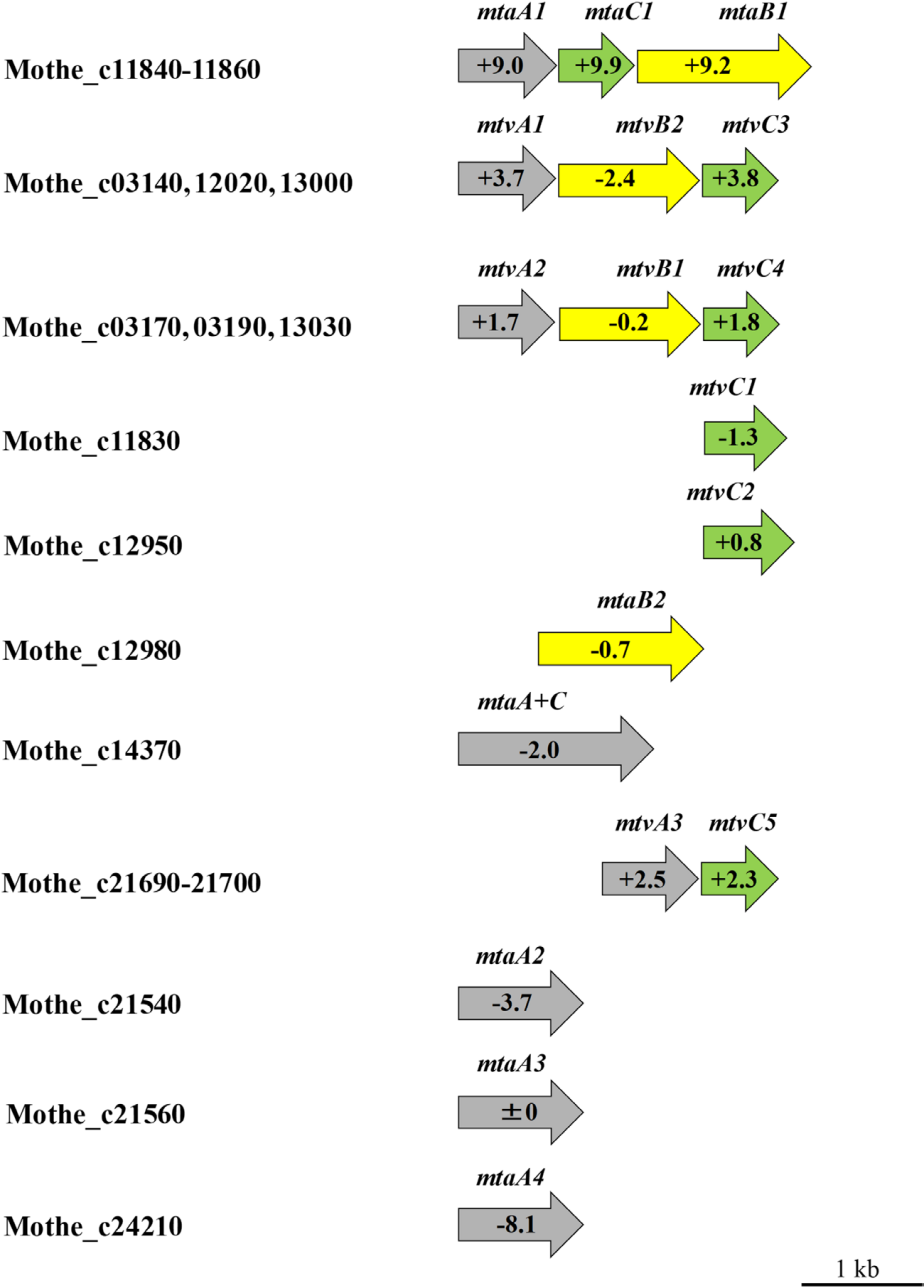
Clusters of methyltransferase encoding genes of *M. thermoacetica* . MTI encoding genes (*mtxB*) are shown in yellow, MTII encoding genes (*mtxA*) are shown in grey and CoP encoding genes (*mtxC*) are represented in green. Numbers indicating log2‐fold changes.

The genome of *M. thermoacetica* encodes three complete methyltransferase systems. Mothe_c11840–11860 (*mtaA1*, *mtaB1* and *mtaC1*) most likely encodes the methanol‐specific methyltransferase system, whereas Mothe_c03140, 12020 and 13000 (*mtv1*, *mtvB2* and *mtvC3*) encode a vanillate‐specific methyltransferase system (Naidu and Ragsdale 2001) and Mothe_c03170, 03190 and 13,030 (*mtvA2*, *mtvB1* and *mtvC4*) a third one with similarity to the vanillate‐specific methyltransferase system. These Mtv methyltransferase systems have a broad substrate specificity and can be used to demethylate several different *O*‐methylated compounds (Daniel et al. 1991; el Kasmi et al. 1994; Naidu and Ragsdale 2001). In addition, three solitary copies of *mtaA* (Mothe_c21540, *mtaA3*; 21,560, *mtaA4* and 24,210, *mtaA5*), one copy of *mtaA* and *mtaC* fused together (Mothe_c14370), one copy of *mtaB* (Mothe_c12980, *mtaB2*), three copies of *mtvC* (Mothe_c11830, *mtvC1*; 12,950, *mtvC2* and 21,700, *mtvC5*) and one copy of *mtvA* (Mothe_c21690, *mtvA3*) can be found in the genome of *M. thermoacetica* (Figure 5). During growth on methanol, the transcript levels of several genes encoding MtvA (*mtvA1, A2 and A3*) and MtvC (*mtvC3, C4 and C5*) were increased in addition to the genes encoding the methanol‐specific methyltransferase system (*mtaA1B1C1*). Interestingly, transcript levels of the solitary copies of *mtaA* were reduced. This indicates that MtaA1 is the methyltransferase used by *M. thermoacetica* to demethylate methyl‐CoP and transfer the methyl group to THF during growth on methanol. The CoP MtaC1 and the MTI MtaB1 are the major proteins involved in channelling the methyl group of methanol into the WLP.

**FIGURE 6:**
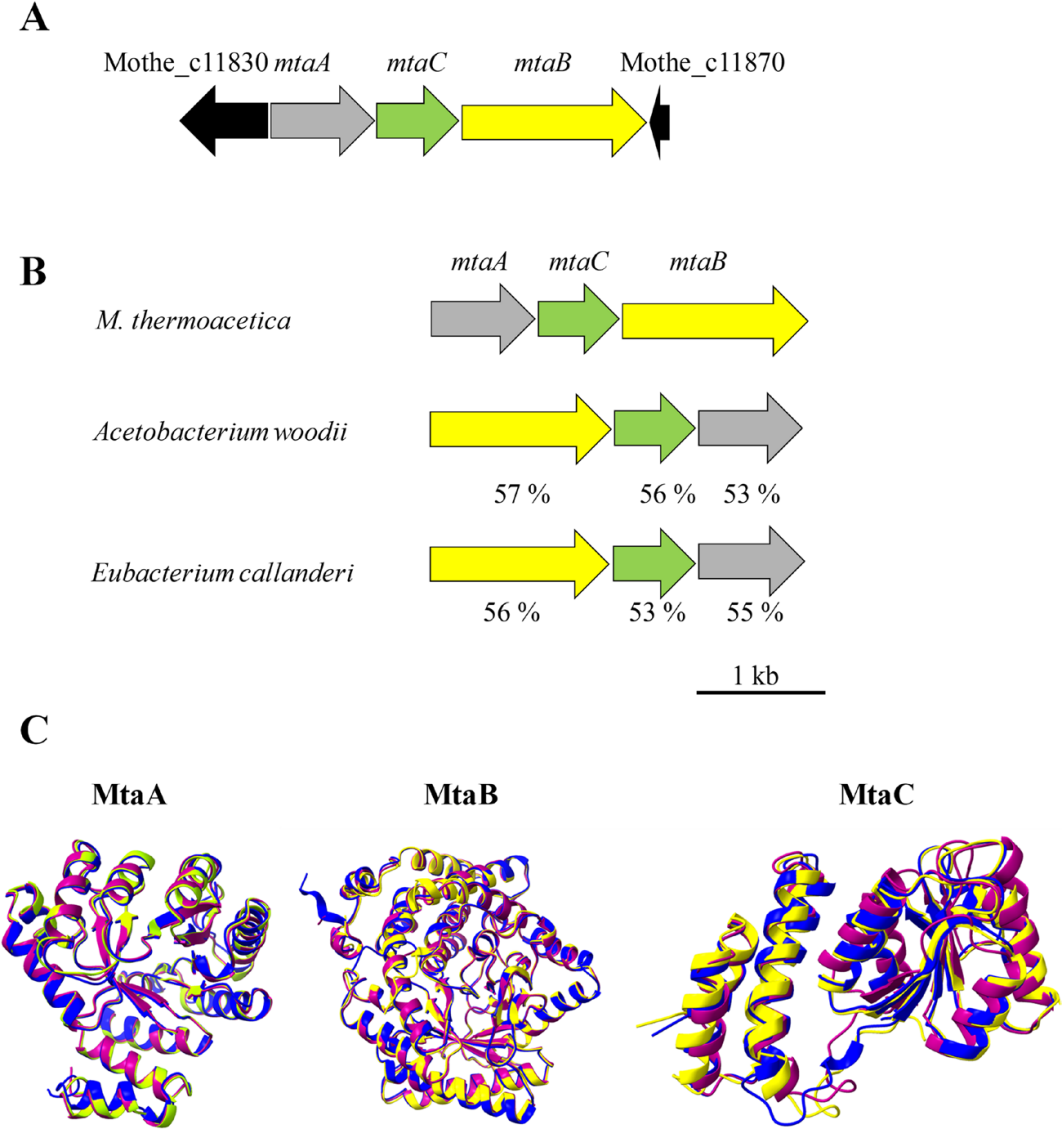
Genetic organisation and comparison of the methanol‐specific methyltransferase system from *M. thermoacetica* to other acetogenic bacteria. Genes, encoding MtaA, MtaB and MtaC are coloured green, yellow or grey, respectively (A). Genes not part of the methanol methyltransferase system are coloured black. The genes *mtaABC* from *M. thermoacetica* were compared to the respective genes in *Acetobacterium woodii* and *Eubacterium callanderi* (B). The identity is given in percentage. A structural overlay of MtaA, MtaB and MtaC from *M. thermoacetica* (blue), *A. woodii* (yellow) and *E. callanderi* (pink) indicates structural similarity (C).

The genetic organisation of the methanol‐specific methyltransferase in *M. thermoacetica* DSM 521 is comparable to that in *M. thermoacetica* ATCC 39073 (Das et al. 2007). The genes *mtaA1B1C1* (Mothe_c11840–11,860) are organized in one cluster. This cluster is flanked upstream by a 675 bp long gene encoding a putative methylmalonyl‐CoA mutase and downstream by a 150 bp long gene encoding a hypothetical protein (Figure 6A). The transcript level of all three genes was highly increased. A putative ribosomal binding region (GGGAGG) was found 8 bp upstream of *mtaA1* and a putative transcriptional terminator was found 12 bp downstream of *mtaB1*. Furthermore, a putative promoter region (ATCGAT‐N_12_‐ATTAAT) was found 27 bp upstream of *mtaA1*. All three genes of the methyltransferase system use ATG as start codon, while the stop codons are TGA, TAA and TAG for *mtaA*, *mtaC* and *mtaB*, respectively. The proteins MtaA (29.7 kDa; 53%–55%), MtaB (52.8 kDa; 56%–57%) and MtaC (22.3 kDa; 53%–56%) are very similar to the methanol‐specific MtaABC from *Acetobacterium woodii* or *Eubacterium callanderi*, respectively (Figure 6B). A structural comparison of each individual subunit of the *M. thermoacetica* methyltransferase system to the respective proteins found in *A. woodii* or *E. callanderi* showed high similarity (Figure 6C).

Co(I) in CoP is required as a supernucleophile (*E*_0_′ = −600 mV) to abstract a methyl cation, but Co(I) tends to spontaneously oxidize to inactive Co(II) (Hagemeier et al. 2006). Therefore, an activating enzyme is required for the reduction of Co(II). Dhaf_2573 (34%), Dhaf_3879 (37%) and Dhaf_4322 (36%) of *Desulfitobacterium hafniense* DBC‐2 or odmC (32%) of *Acetobacterium dehalogenans* encode such an activating enzyme (Schilhabel et al. 2009; Studenik et al. 2011). The homologue with the highest similarity to these found in *M. thermoacetica* is Mothe_c11760. The transcript level of Mothe_c11760 was increased in methanol‐grown cells by a log2FC of + 1.9. Therefore, we postulate that Mothe_c11760 encodes the activating enzyme in *M. thermoacetica* . This suggestion is in line with the findings by Schilhabel et al. (2009) who suggested Mothe_c11760 as gene encoding an activating enzyme in *M. thermoacetica* based on its similarity to odmC from *A. dehalogenans*.

### 2.7 Enzyme Activities of Cell‐Free Extracts Prepared From Cells Grown on Methanol in Presence or Absence of DMSO

To address a possible differential regulation of enzyme activities, cell‐free extracts were prepared from cells grown on glucose (CE_Glu_), methanol (CE_MeOH_) or methanol + DMSO (CE_Me+DMSO_) and enzyme activities were measured (Table 1). The electron‐bifurcating hydrogenase activity was 5–10‐fold lower in CE_MeOH_ or CE_Me+DMSO_ compared to CE_Glu_. In contrast, the NADP^+^‐reducing hydrogenase activity was 13 or 23‐fold higher in CE_MeOH_ or CE_Me+DMSO_ compared to CE_Glu_, respectively. This suggests that in cells growing on methanol or methanol + DMSO the NADP^+^‐reducing hydrogenase is the only active soluble hydrogenase. This finding is in line with the transcriptomic data. To clarify whether the NADP^+^‐reducing hydrogenase is involved in hydrogen formation or in hydrogen oxidation, the NADP^+^‐dependent hydrogenase was purified as previously described (Rosenbaum and Müller 2024). The purified enzyme catalysed H_2_‐dependent NADP^+^ reduction with an activity of 8.3 U/mg. To test for NADPH‐dependent hydrogen formation, 100 μg of the purified hydrogenase was incubated in buffer at 60°C in a 1‐ml total volume using 8.7‐ml anoxic serum bottles. The reaction was started by adding 30 mM NADPH, but hydrogen formation was not observed. Thus, it can be assumed that the NADP^+^‐reducing hydrogenase is not involved in hydrogen production, but rather oxidizes the hydrogen produced. As mentioned before, Fdh‐Ech may be responsible for the hydrogen production observed during growth and resting cell experiments.

**TABLE 1:** Activities of WLP enzymes, hydrogenase, formate dehydrogenase and DMSO reductase in cell‐free extract of *M. thermoacetica* grown on glucose, methanol or methanol + DMSO.

Enzyme	Reaction catalysed	Activity (U/mg)^a^ in cells grown on		
		Glucose^b^	Methanol	Methanol + DMSO
NADP^+^‐reducing hydrogenase	H_2_ + NADP^+^ → NADPH	0.07 ± 0.03	0.89 ± 0.05	1.61 ± 0.09
Electron‐bifurcating hydrogenase	H_2_ + NAD^+^ + Fd → NADH + Fd^2−^	0.20 ± 0.02	0.02 ± 0.01	0.04 ± 0.02
CO‐DH	CO + Fd → Fd^2−^	0.39 ± 0.06	2.20 ± 0.51	1.00 ± 0.13
NADP^+^‐dependent formate dehydrogenase	Formate + NADP^+^ → NADPH	0.32 ± 0.03	1.55 ± 0.06	1.30 ± 0.06
DMSO reductase	BV^2−^ + DMSO → BV + DMS	0.03 ± 0.01	0.02 ± 0.01	0.26 ± 0.02

^a^ Enzyme activities were determined as described in Material and methods. All values are mean ± SD; *n* = 3.

^b^ Values are taken from (Rosenbaum et al. 2026).

Next, we measured formate‐dependent NADP^+^ reduction. The activity was 5 or 4‐fold higher in CE_MeOH_ or CE_Me+DMSO_ compared to CE_Glu_, respectively. The CO‐dependent ferredoxin reduction was 7‐ or 3‐fold higher in CE_MeOH_ or CE_Me+DMSO_ compared to CE_Glu_, respectively. This is consistent with the transcriptomic data and since the activity of the CO‐DH/ACS is the only source of acetyl‐CoA, the precursor of every biosynthetic pathway, the increased CO‐DH activity was not unexpected.

Last, we measured DMSO reductase activity, which was highest in CE_Me+DMSO_ with a BV:DMSO oxidoreductase activity of 0.26 U/mg. The activity in CE_Glu_ or CE_MeOH_ was 8‐ to 12‐fold lower, respectively, indicating a strong induction of DMSO reduction by DMSO.

## 3 Discussion

Methanol and methylated compounds are used by many acetogenic bacteria due to their high natural abundance (Drake et al. 2008). Previous studies have examined methanol metabolism in several acetogens (Kremp and Müller 2021). Compared to other C1 substrates, the major difference is the need for a methyltransferase system to funnel the methyl group into the WLP (Kremp and Müller 2021).

Our data clearly demonstrate that the methanol‐specific methyltransferase system is encoded by *mtaA1B1C1* (Mothe_c11840 to 11870). In contrast, Das et al. (2007) suggested Mothe_c24210 to be the MTII involved in methanol metabolism, but this could not be confirmed by our transcriptome analyses. Indeed, Mothe_c24210 encodes a MtaA methyltransferase, but this methyltransferase is not involved in methanol metabolism since the transcript count for the encoding gene was highly reduced (log2FC = −8.1). Furthermore, our transcriptome analyses could confirm Mothe_c11850 and Mothe_c11860 as the CoP and MTI, respectively, involved in methanol metabolism, which is in line with the findings by Das et al. (2007). The upregulation of other methyltransferase systems by methanol is not unusual (Kremp et al. 2018), but reflects an unspecificity in the regulation of the expression system rather than on the protein level.

During methyl‐group oxidation, methyl‐THF is first oxidized to methylene‐THF by the MTHFR. This step is thermodynamically challenging because the redox potential of methylene‐THF/methyl‐THF (*E*_0_′ = −200 mV) does not allow for the reduction of NAD^+^/NADH (*E*_0_′ = −320 mV) (Thauer et al. 1977). Indeed, the methyl‐THF‐dependent NAD^+^ reduction is highly endergonic (Δ*G*_0_′ = +23 kJ/mol) (Öppinger et al. 2022). There are four different types of MTHFR known in bacteria (Öppinger et al. 2022). These differ both in structural complexity and the electron carrier involved: either NAD^+^, Fd or unknown electron carriers. *A. woodii* uses a type III MTHFR with NAD^+^ as electron carrier. This bacterium overcomes the thermodynamic barrier by lowering the intracellular concentration of methylene‐THF via a higher methylene dehydrogenase activity (Bertsch et al. 2015). *M. thermoacetica* uses a type IV MTHFR which has the most complex subunit composition (Mock et al. 2014). Based on genetic data and biochemical evidence this type of MTHFR consists of heterodisulfide reductase‐like subunits HdrABC, the reductase MetVF and the adapter protein MvhD. HdrA has been shown to be electron‐bifurcating in methanogenic archaea (Kaster et al. 2011; Ramos et al. 2015; Appel et al. 2021). Therefore, it is suggested that the MTHFR from *M. thermoacetica* might also be electron‐bifurcating. Thauer and colleagues identified NADH as one of the electron carriers used by the MTHFR (Mock et al. 2014) and it was suggested that the second electron carrier beside methylene‐THF might be a membrane‐bound electron carrier such as menaquinone or cytochrome *b* since ferredoxin was not used as electron carrier (Das et al. 1989; Mock et al. 2014). We recently proposed a different mechanism: reduced menaquinone (*E*_0_′ = −74 mV) serves as electron donor for methylene‐THF reduction (methylene‐THF/methyl‐THF, *E*_0_′ = −200 mV), an endergonic reaction driven by electron confurcation with NADH (*E*_0_′ = −320 mV) as third reactant. In the reverse direction, as occurs during methanol metabolism, the oxidation of methyl‐THF coupled to the reduction of MQ is exergonic and drives the endergonic reduction of NAD^+^.

**FIGURE 7:**
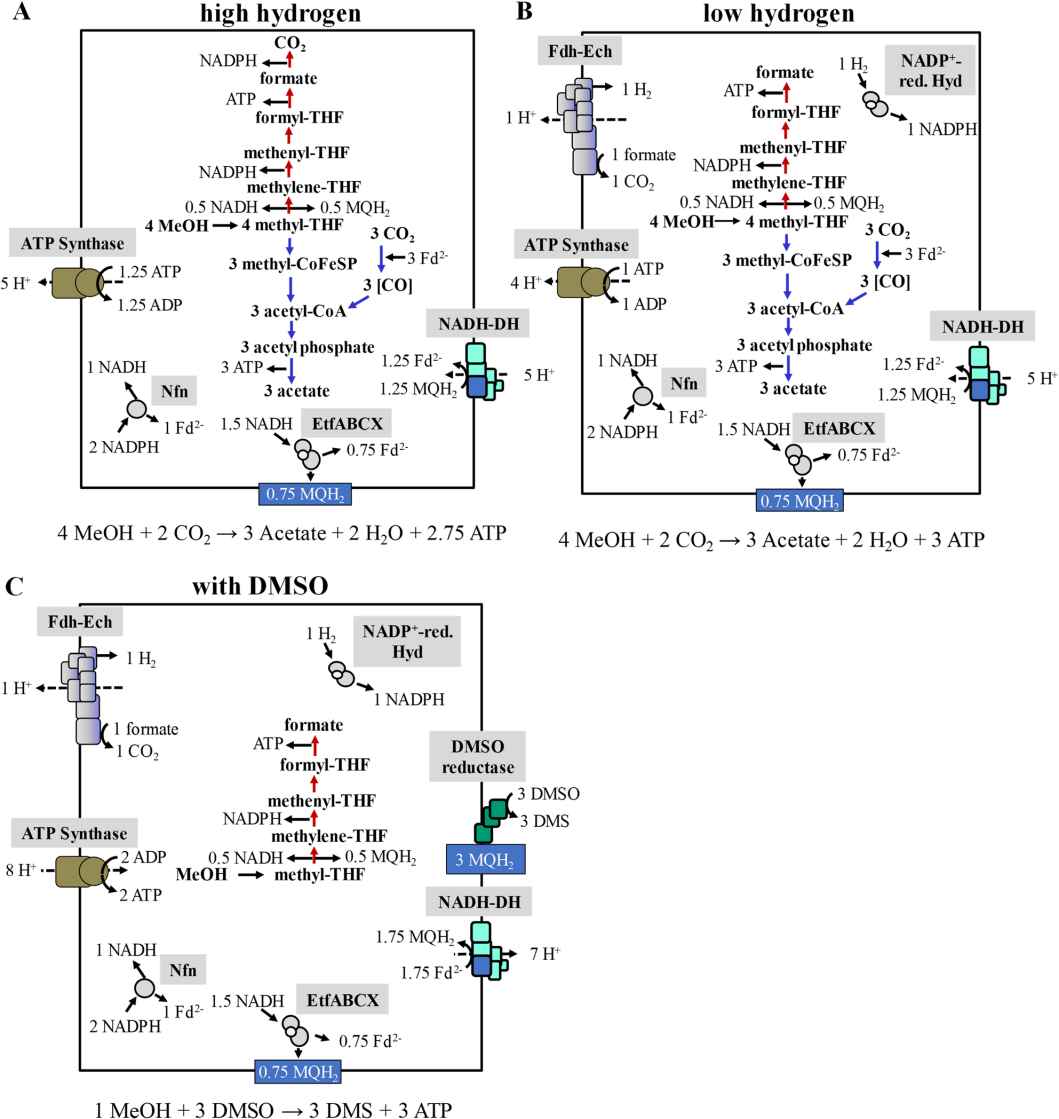
Model of methanol metabolism in *M. thermoacetica* . Methanol metabolism in *M. thermoacetica* at high (A) and low hydrogen concentration (B) or in presence of DMSO (C). For further information, see text.

In summary during methanol metabolism, 1 mol of methyl‐THF is oxidized to CO_2_, thereby producing 1 mol of ATP and 2 mol of NADPH, 0.5 mol of NADH and menaquinol (MQH_2_). To reduce CO_2_ in the carbonyl branch by the CO‐DH/ACS 3 mol of reduced ferredoxin are required. Several enzymes are involved in transferring electrons from NADPH, NADH and MQH_2_ to ferredoxin (Huang et al. 2012; Rosenbaum et al. 2021). First, 2 mol of NADPH are oxidized by the Nfn and the electron carriers NAD^+^ and ferredoxin are reduced at a ratio of 2:1:1 (Huang et al. 2012). Next, the EtfABCX complex uses 1.5 mol of NADH to reduce 0.75 mol of ferredoxin and MQH_2_ in an electron‐bifurcating reaction (Ledbetter et al. 2017; Feng et al. 2021; Rosenbaum et al. 2021). The NADH dehydrogenase, fueled by the proton gradient then transfers electrons from MQH_2_ to ferredoxin, resulting in a further 1.25 mol of reduced ferredoxin. Overall, 3 mol of ferredoxin are reduced and serve as an electron donor for the CO‐DH/ACS. The required electrochemical ion potential is established at the expense of 1.25 ATP by the ATP synthase. With 3 mol of enzyme‐bound CO available, acetyl‐CoA can now be synthesised by condensing them with 3 mol of methyl‐CoFeSP generated in the methyl branch. The formation of acetate from acetyl‐CoA yields 3 mol of ATP. In total, 4 mol of methanol and 2 mol of CO_2_ are metabolised to 3 mol of acetate, with 0.92 ATP/acetate or 0.68 ATP/methanol formed (Figure 7A). In this scheme, hydrogen is not produced making it a likely metabolism under relatively high environmental hydrogen concentrations, because formate‐dependent hydrogen formation (Δ*G*_0_′ = −3.5 kJ/mol) by the Fdh‐Ech complex is a rather weak exergonic reaction and becomes thermodynamical unfavourable at a high hydrogen to formate ratio.

The picture changes at relatively low hydrogen to formate ratio (Figure 7B). Under these conditions, the Fdh‐Ech complex catalyses exergonic electron transfer from formate to protons, resulting in hydrogen production and generation of an electrochemical ion gradient. This hypothesis is supported by the detection of hydrogen formation during physiological experiments, as well as by genome‐wide transcription analyses. The produced hydrogen is subsequently oxidized by the NADP^+^‐reducing hydrogenase, whose activity is increased in methanol‐grown cells (Rosenbaum and Müller 2024). In contrast, the activity of the electron‐bifurcating hydrogenase was reduced during growth on methanol. We hypothesise that it is crucial for the microorganism to change the hydrogenase used from the electron‐bifurcating hydrogenase to the NADP^+^‐reducing hydrogenase for maintaining a low H_2_ partial pressure, favouring the exergonic formate‐dependent hydrogen production by the Fdh‐Ech complex. This hypothesis is supported by thermodynamics; during growth on methanol, high amounts of reducing equivalents such as NADH and reduced ferredoxin are available. These electron carriers can be easily oxidized in an exergonic reaction by the electron‐bifurcating/confurcating hydrogenase from *M. thermoacetica* leading to the formation of hydrogen (Wang et al. 2013), whereas the NADP^+^‐reducing hydrogenase lacks this capability.

In total, 2 mol of NADPH, 0.5 mol of NADH and MQH_2_ become available to reduce ferredoxin. This allows the reduction of 3 mol of ferredoxin, which are required to reduce three CO_2_ to CO by the CO‐DH/ACS. The Nfn, EtfABCX and NADH dehydrogenase are again involved in maintaining redox carrier balancing. However, only 1 mol of ATP is required for proton translocation, since the Fdh‐Ech complex contributes to the electrochemical proton gradient. As a result, *M. thermoacetica* conserves slightly more ATP and now thrives on 1 ATP/acetate or 0.75 ATP/methanol. Methanol oxidation is favoured at low hydrogen concentrations.

One remarkable feature of *M. thermoacetica* is its ability to reduce alternative electron acceptors such as DMSO or nitrate (Seifritz et al. 1993; Rosenbaum et al. 2022). The growth of *M. thermoacetica* on methanol was stimulated by DMSO while acetate formation was shut off. While the overall carbon flow scheme remains similar, the electron flow changes. The DMSO reductase accepts electrons from menaquinone; therefore, all electrons must be funnelled to menaquinone. This is achieved via the EtfABCX complex and the “headless” NADH dehydrogenase (Figure 7C). Thereby, no acetate is formed and 3 mol of ATP are synthesised per mol of methanol oxidized; this is more than 300% higher ATP yield than with methanol alone! This increased ATP yield may be of benefit when ATP‐demanding products shall be produced from methanol in *M. thermoacetica* . Moreover, it is noteworthy that methanol is completely oxidized to CO_2_ in the presence of DMSO. The same was observed in *A. woodii* in the presence of the alternative electron acceptor caffeate (Litty et al. 2022). When the formate dehydrogenase HDCR was deleted, methanol was completely oxidized to formate (Moon et al. 2023). Formatogenesis from methanol would also be an interesting biotechnological perspective for *M. thermoacetica* .

## 4 Materials and Methods

### 4.1 Organism and Cultivation

*Moorella thermoacetica* DSM 521 was cultivated under anoxic conditions at 55°C in bicarbonate‐buffered or phosphate‐buffered complex medium and grown in 120 mL serum bottles (Glasgerätebau Ochs, Bovenden/Lenglern, Germany) filled with 50 mL complex medium, supplemented with 0–1.1 M methanol under an N_2_ + CO_2_ (80/20 [v/v]) or 100% N_2_ atmosphere (Rosenbaum et al. 2021). Routinely, 60 mM methanol was used as a substrate. DMSO was added at a concentration of 40 mM. The media were prepared according to the anaerobic techniques described previously by (Hungate 1969; Bryant 1972).

### 4.2 Resting Cell Experiments

Cells were grown in bicarbonate‐containing complex medium in 1 L flasks (Schott AG, Mainz, Germany) filled with 500 mL medium using 60 mM methanol as carbon and energy source in the presence or absence of 20 mM DMSO. Resting cells were prepared and experiments were performed as described previously (Rosenbaum et al. 2022).

### 4.3 Preparation of Cell‐Free Extract

*M. thermoacetica* was cultivated as described above using 60 mM methanol as carbon‐ and energy source in the presence or absence of 20 mM DMSO. Cell‐free extract was prepared as described previously (Rosenbaum et al. 2022).

### 4.4 Analytical Methods

Metabolite analyses were carried out using high‐pressure liquid chromatography and gas chromatography as described previously (Schwarz and Müller 2020).

### 4.5 Purification of the NADP ^+^‐Reducing Hydrogenase From *M. thermoacetica*

The NADP^+^‐reducing hydrogenase from *M. thermoacetica* was purified as described previously (Rosenbaum and Müller 2024).

### 4.6 Enzyme Activity Assays

All enzyme assays were carried out at 55°C in 1.8 mL anoxic cuvettes (Glasgerätebau Ochs, Bovenden/Lenglern, Germany) filled with enzyme buffer (50 mM Tris/HCl (pH 8), 10 mM NaCl, 2 mM DTE and 4 μM resazurin) with a final volume of 1 mL. One unit is defined as the transfer of 2 μmol electrons min^−1^. All measurements were performed in biological replicates using 50 to 150 μg of protein. NAD(P)^+^/NAD(P)H was monitored spectrophotometrically at 340 nm (*ε* = 6.3 mM^−1^ cm^−1^), ferredoxin (Fd) (isolated from *Clostridium pasteurianum* (Schönheit et al. 1978)) at 430 nm (*ε* = 13.1 mM^−1^ cm^−1^) or benzyl viologen (BV) at 600 nm (*ε* = 12 mM^−1^ cm^−1^) using concentrations of 30 μM, 1 and 2 mM. FDH activity was measured under a N_2_ atmosphere (1 × 10^5^ Pa), CO‐DH under a CO atmosphere (1 × 10^5^ Pa) and hydrogenase activity under a H_2_ atmosphere (1 × 10^5^ Pa). The formate‐dependent reactions were started by adding 20 mM formate, H_2_‐dependent reactions were started by adding H_2_ and CO‐dependent reactions were started by adding CO. For DMSO reductase activity BV was first reduced with sodium dithionite and the reaction was started by addition of 20 mM DMSO (*n* = 3; SD). Hydrogen formation from NADPH was measured in 8.7 mL anoxic serum bottles filled with buffer (50 mM Tris/HCl (pH 8.0), 10 mM NaCl, 2 mM DTE, 4 μM resazurin) at a final volume of 1 mL under a 100% N_2_ gas atmosphere and 60°C. For the enzyme assay 100 μg of protein and 30 mM NADPH was used. Hydrogen production was measured using gas chromatography as described previously (Schwarz and Müller 2020).

### 4.7 Sampling and Sequencing of RNA

The transcriptomes of *M. thermoacetica* DSM 521 growing on methanol or methanol + DMSO were compared to the transcriptome of glucose‐ or methanol‐grown cells. Cells were cultivated in biological triplicates and harvested in the exponential growth phase (methanol: OD_600_ = 0.35; methanol + DMSO: OD_600_ = 0.4). Harvested cells were resuspended in 800 μL RLT buffer (RNeasy Mini Kit, Qiagen) with β‐mercaptoethanol (10 μL ml^1^) and cell lysis was performed using a laboratory ball mill. Subsequently 400 μL RLT buffer (RNeasy Mini Kit Qiagen) with β‐mercaptoethanol (10 μL/mL) and 1200 μL 96% [v/v] ethanol were added. For RNA isolation, the RNeasy Mini Kit (Qiagen) was used as recommended by the manufacturer, but instead of RW1 buffer RWT buffer (Qiagen) was also used in order to isolate RNAs smaller than 200 nt. To determine the RNA integrity number (RIN), the isolated RNA was run on an Agilent Bioanalyzer 2100 using an Agilent RNA 6000 Nano Kit as recommended by the manufacturer (Agilent Technologies, Waldbronn, Germany). Remaining genomic DNA was removed by digesting with TURBO DNase (Invitrogen, ThermoFischer Scientific, Paisley, United Kingdom). The Illumina Ribo‐Zero plus rRNA Depletion Kit (Illumina, San Diego, CA, USA) was used to reduce the amount of rRNA‐derived sequences. For sequencing, the strand‐specific cDNA libraries were constructed with a NEBNext Ultra II directional RNA library preparation kit for Illumina (New England BioLabs, Frankfurt am Main, Germany). To assess quality and size of the library samples were run on an Agilent Bioanalyzer 2100 using an Agilent High Sensitivity DNA Kit as recommended by the manufacturer (Agilent Technologies, Waldbronn, Germany). Concentration of the libraries was determined using the Qubit dsDNA HS Assay Kit as recommended by the manufacturer (Life Technologies GmbH, Darmstadt, Germany). Sequencing was performed on the NovaSeq X Plus instrument (Illumina) using NovaSeq X Series 1.5B Reagent Kit (100 cycles) for sequencing in the paired‐end mode and running 2 50 cycles. After processing of the 50 bp single‐end raw reads with trimmomatic (version 0.39) (Bolger et al. 2014), Salmon (v 1.10.2) (Patro et al. 2017) was used for mapping of the trimmed paired‐end read against the genome of *M. thermoacetica* DSM521 (Poehlein et al. 2015). A file containing all annotated transcripts (without rRNA genes) and the whole genome as decoy was prepared with a k‐mer size of 11 as mapping backbone. Decoy‐aware mapping was done in selective‐alignment mode with ‘‐‐mimicBT2’, ‘‐‐disableChainingHeuristic’ and ‘‐‐recover‐Orphans’ flags as well as sequence and position bias correction. For ‐‐fldMean and ‐‐fldSD, a value of 325 and 25 was used, respectively. Salmon's quants files were subsequently loaded into R (v 4.4.2) (R Core Team 2020) using the tximport package (v 1.32.0) (Soneson et al. 2015). Normalisation of the reads was done with DeSeq2 (v 1.44.0) (Love et al. 2014) and foldchange‐shrinkages were calculated with DeSeq2 and the apeglm package (v 1.26.1) (Zhu et al. 2019). Genes with a log2‐fold change of +2/−2 and a *p*‐adjust value of < 0.05 were considered differentially expressed.

### 4.8 Analysis of Transcriptomic Data and Bioinformatic Analysis

Genes were also manually analysed and curated using the KEGG, KO Database, the Integrated Microbial Genomes‐Expert Review (IMG‐ER) database, InterPro, TMHMM and BLAST.

### 4.9 Replicates, Statistical Analysis and Data Representation

All experiments were performed with at least three independent biological replicates. Each biological replicate was initiated with a freshly prepared medium and inoculum. Each biological replicate included three technical duplicates. Data from one representative biological replicate are presented in the figures. Shown values represent means with standard deviation (SD).

## Author Contributions

**Florian P. Rosenbaum:** writing – original draft, investigation, conceptualization, data curation, writing – review and editing, formal analysis. **Anja Poehlein:** data curation, investigation, formal analysis, writing – review and editing. **Volker Müller:** writing – original draft, conceptualization, writing – review and editing. **Rolf Daniel:** investigation, writing – review and editing, formal analysis, data curation.

## Funding

This work was supported by Deutsche Forschungsgemeinschaft.

## Conflicts of Interest

The authors declare no conflicts of interest.

## Supporting information


**Figure S1:** DMSO is used as alternative electron acceptor by *M. thermoacetica* . Cells were cultivated in 5 mL phosphate‐buffered medium under a 100% N_2_ atmosphere at 55°C with 60 mM methanol as substrate in the presence (♦) or absence (◊) of DMSO and the OD_600_ was monitored over time (SD, *n* = 3).
**Table S1:** The most upregulated genes of *M. thermoacetica* during growth on methanol.
**Table S2:** The most downregulated genes of *M. thermoacetica* during growth on methanol.
**Table S3:** The most upregulated genes of *M. thermoacetica* during growth on methanol + DMSO.
**Table S4:** The most downregulated genes of *M. thermoacetica* during growth on methanol + DMSO.

## Data Availability

Transcriptome data have been deposited in the National Center for Biotechnology Information (NCBI) Sequence Read Archive (SRA) as BioProject PRJNA1358060. All other data of this study are available from the corresponding author upon reasonable request.
