# Supplementary material for: Methanol Metabolism in the Cytochrome and Menaquinone‐Containing Acetogen Moorella thermoacetica
Source: Environ Microbiol. 2026 Jul 22;28(7):e70377. doi: 10.1111/1462-2920.70377 (PMC13391211; doi:10.1111/1462-2920.70377)
Supplement: Supplementary file 1 — Figure S1: DMSO is used as alternative electron acceptor by M. thermoacetica . Cells were cultivated in 5 mL phosphate‐buffered medium under a 100% N2 atmosphere at 55°C with 60 mM methanol as substrate in the presence (♦) or absence (◊) of DMSO and the OD600 was monitored over time (SD, n = 3). Table S1: The most upregulated genes of M. thermoacetica during growth on methanol. Table S2: The most downregulated genes of M. thermoacetica during growth on methanol. Table S3: The most upregulated genes of M. thermoacetica during growth on methanol + DMSO. Table S4: The most downregulated genes of M. thermoacetica during growth on methanol + DMSO. [file EMI-28-e70377-s001.docx]

**Supplementary Information**

**for the manuscript:**

**Methanol metabolism in the cytochrome and menaquinone-containing acetogen *Moorella thermoacetica***

Florian P. Rosenbaum^1^, Anja Poehlein^2^, Rolf Daniel^2^, Volker Müller^1*^

*^1^Department of Molecular Microbiology & Bioenergetics, Institute of Molecular Biosciences, Johann Wolfgang Goethe University, 60438 Frankfurt am Main, Germany*

*^2^Genomic and Applied Microbiology & Göttingen Genomics Laboratory, Georg-August University Göttingen, 37077 Göttingen, Germany*

^*^correspondence address: Prof. Volker Müller. Department of Molecular Microbiology & Bioenergetics, Institute of Molecular Biosciences, Johann Wolfgang Goethe University, Frankfurt am Main, Germany; Phone: 49-6979829507; Fax: 49-69-79829306;

E-mail: [vmueller@bio.uni-frankfurt.de](mailto:vmueller@bio.uni-frankfurt.de)

**Supplementary figure 1**

**Supplementary table 1**

**Supplementary table 2**

**Supplementary table 3**

**Supplementary table 4**

**Supplementary figures**

**Supplementary figure 1**

**Figure S1. DMSO is used as alternative electron acceptor by *M. thermoacetica*.** Cells were cultivated in 5 ml phosphate-buffered medium under a 100 % N_2_ atmosphere at 55°C with 60 mM methanol as substrate in the presence (♦) or absence (◊) of DMSO and the OD_600_ was monitored over time (SD, n = 3).

**Supplementary tables**

**Supplementary table 1: The most upregulated genes of *M. thermoacetica* during growth on methanol.**

| **gene** | **annotation** | **substrate** | | **Log_2_ (fold change)** |
| --- | --- | --- | --- | --- |
|  |  | **methanol** | **glucose** |  |
| MOTHE_c19190 | NADP-reducing hydrogenase subunit HydD | 1.400 | 0 | 14.5 |
| MOTHE_c23350 | fructose-1,6-bisphosphatase | 20.092 | 8 | 11.3 |
| MOTHE_c19210 | NADP-reducing hydrogenase subunit HydC | 1.757 | 1 | 10.2 |
| MOTHE_c11850 | corrinoid protein CoP MtaC1 | 95.478 | 101 | 9.9 |
| MOTHE_c19180 | NADP-reducing hydrogenase subunit HydA | 7.963 | 10 | 9.6 |
| MOTHE_c07320 | flagellar biosynthetic protein FliQ | 89 | 0 | 9.3 |
| MOTHE_c19200 | NADP-reducing hydrogenase subunit HydE | 1.453 | 2 | 9.3 |
| MOTHE_c11860 | methyl-THF:CoP methyltransferases MtaB | 165.010 | 283 | 9.2 |
| MOTHE_c11840 | methyltransferases MtaA | 94.813 | 181 | 9.0 |
| MOTHE_c19220 | NADP-reducing hydrogenase subunit HydB | 11.482 | 20 | 9.0 |
| MOTHE_c12160 | putative pit accessory protein | 54 | 0 | 8.6 |
| MOTHE_c24510 | stage III sporulation protein D | 59 | 0 | 8.5 |
| MOTHE_c15240 | stage III sporulation protein AE precursor | 1.482 | 5 | 8.3 |
| MOTHE_c07440 | TPR repeat-containing protein YrrB | 132 | 0 | 7.8 |
| MOTHE_c00440 | peptidoglycan-N-acetylmuramic acid deacetylase PdaA precursor | 491 | 2 | 7.7 |
| MOTHE_c07990 | RNA polymerase sigma-F factor | 195 | 1 | 7.6 |
| MOTHE_c13040 | stage IV sporulation protein A | 6.394 | 34 | 7.6 |
| MOTHE_c13590 | SpoVA protein | 32 | 0 | 7.5 |
| MOTHE_c07970 | sporulation sigma-E factor-processing peptidase | 798 | 4 | 7.5 |
| MOTHE_c19230 | NADP-reducing hydrogenase subunit HydF | 5.916 | 37 | 7.3 |
| MOTHE_c05610 | tagatose-6-phosphate kinase | 6.703 | 41 | 7.3 |
| MOTHE_c13940 | transcriptional regulator MntR | 217 | 1 | 7.2 |
| MOTHE_c00810 | PRC-barrel domain protein | 1.893 | 16 | 6.9 |
| MOTHE_c15260 | stage III sporulation protein AC/AD protein family protein | 22 | 0 | 6.7 |
| MOTHE_c05620 | D-ribose-binding periplasmic protein precursor | 7.406 | 72 | 6.7 |
| MOTHE_c07980 | RNA polymerase sigma-E factor precursor | 534 | 6 | 6.5 |
| MOTHE_c04500 | PrkA AAA domain protein | 2.797 | 29 | 6.5 |
| MOTHE_c08010 | PRC-barrel domain protein | 18 | 0 | 6.4 |
| MOTHE_c15270 | stage III sporulation protein SpoAB | 664 | 8 | 6.3 |
| MOTHE_c00540 | cell wall-binding protein YocH precursor | 495 | 6 | 6.2 |
| MOTHE_c15250 | stage III sporulation protein AC/AD protein family protein | 192 | 3 | 6.1 |
| MOTHE_c15230 | stage III sporulation protein AF | 1.055 | 16 | 6.1 |
| MOTHE_c08360 | calcium-transporting ATPase | 400 | 6 | 6.0 |
| MOTHE_c19050 | sporulation membrane protein YtrH | 44 | 1 | 6.0 |
| MOTHE_c15210 | stage III sporulation protein AH | 1.147 | 17 | 6.0 |
| MOTHE_c13780 | putative manganese catalase | 69 | 1 | 5.9 |
| MOTHE_c09490 | F420-0:gamma-glutamyl ligase | 246 | 4 | 5.9 |
| MOTHE_c07330 | flagellar biosynthetic protein FliR | 669 | 11 | 5.9 |
| MOTHE_c18810 | hypoxic response protein 1 | 461 | 8 | 5.9 |
| MOTHE_c07430 | flagellar basal-body rod protein FlgG | 893 | 16 | 5.8 |
| MOTHE_c08350 | calcium-transporting ATPase | 1.100 | 21 | 5.8 |
| MOTHE_c19630 | D-glycero-alpha-D-manno-heptose 1-phosphate guanylyltransferase | 32.498 | 608 | 5.7 |
| MOTHE_c15200 | phenylalanine-specific permease | 496 | 10 | 5.6 |
| MOTHE_c09480 | N-acetylmuramoyl-L-alanine amidase LytC precursor | 545 | 12 | 5.4 |
| MOTHE_c05590 | D-tagatose-1,6-bisphosphate aldolase subunit KbaY | 14.449 | 27 | 5.3 |
| MOTHE_c05390 | YabP family protein | 478 | 12 | 5.3 |
| MOTHE_c05260 | stage II sporulation protein P (SpoIIP) | 425 | 11 | 5.2 |
| MOTHE_c06030 | teichoic acids export ATP-binding protein TagH | 336 | 9 | 5.2 |
| MOTHE_c11080 | stage V sporulation protein B | 378 | 10 | 5.2 |
| MOTHE_c05580 | HTH-type transcriptional repressor GlcR | 504 | 14 | 5.1 |
| MOTHE_c07180 | Yop proteins translocation protein L | 1.323 | 39 | 5.1 |
| MOTHE_c24520 | L-Ala--D-Glu endopeptidase precursor | 1.067 | 32 | 5.1 |
| MOTHE_c05600 | bifunctional phosphoglucose/phosphomannose isomerase | 1.060 | 32 | 5.0 |
| MOTHE_c07390 | RNA polymerase sigma-D factor | 551 | 19 | 4.8 |
| MOTHE_c07420 | flagellar basal-body rod protein FlgG | 948 | 33 | 4.8 |
| MOTHE_c07150 | flagellar hook-basal body complex protein FliE | 164 | 5 | 4.8 |
| MOTHE_c13560 | 2-hydroxyglutaryl-CoA dehydratase. D-component | 247 | 8 | 4.8 |
| MOTHE_c17290 | multifunctional cyclase-dehydratase-3-O-methyl transferase TcmN | 2.243 | 80 | 4.8 |
| MOTHE_c07200 | flagellar FliJ protein | 507 | 18 | 4.8 |
| MOTHE_c17310 | phosphomethylpyrimidine synthase | 17.537 | 116 | 4.8 |
| MOTHE_c07290 | flagellar motor switch protein FliN | 230 | 8 | 4.8 |
| MOTHE_c07170 | flagellar motor switch protein FliG | 1.592 | 59 | 4.8 |
| MOTHE_c07410 | YceG-like family protein | 550 | 20 | 4.7 |
| MOTHE_c07340 | flagellar biosynthetic protein FlhB | 937 | 36 | 4.7 |
| MOTHE_c13020 | methyl-THF:CoP methyltransferases MtvB4 | 3.408 | 134 | 4.7 |
| MOTHE_c07160 | flagellar M-ring protein | 2.572 | 104 | 4.6 |
| MOTHE_c17950 | serine dehydrogenase proteinase | 19 | 0 | 4.6 |
| MOTHE_c07280 | flagellar FliL protein | 220 | 9 | 4.6 |
| MOTHE_c07270 | motility protein B | 440 | 18 | 4.6 |
| MOTHE_c05400 | putative stage IV sporulation protein YqfD | 386 | 17 | 4.5 |
| MOTHE_c24130 | IS66 Orf2 like protein | 189 | 8 | 4.5 |
| MOTHE_c07350 | flagellar biosynthesis protein FlhA | 2.386 | 107 | 4.5 |
| MOTHE_c17320 | phosphomethylpyrimidine synthase | 2.443 | 106 | 4.5 |
| MOTHE_c05630 | ribose import ATP-binding protein RbsA | 2.634 | 120 | 4.5 |
| MOTHE_c05640 | ribose transport system permease protein RbsC | 1.099 | 53 | 4.4 |
| MOTHE_c08000 | stage II sporulation protein R | 144 | 7 | 4.4 |
| MOTHE_c22940 | transposase | 44 | 2 | 4.3 |
| MOTHE_c18830 | glycogen synthase | 3.088 | 154 | 4.3 |
| MOTHE_c12990 | putative sulfoacetate transporter SauU | 158 | 8 | 4.3 |
| MOTHE_c18820 | glycogen phosphorylase | 5.043 | 254 | 4.3 |
| MOTHE_c17300 | nicotinate-nucleotide--dimethylbenzimidazole phosphoribosyltransferase | 3.082 | 154 | 4.3 |
| MOTHE_c22930 | integrase core domain protein | 580 | 30 | 4.3 |
| MOTHE_c22740 | putative permease | 328 | 17 | 4.2 |
| MOTHE_c07370 | flagellum site-determining protein YlxH | 1.027 | 56 | 4.2 |
| MOTHE_c06080 | putative glycosyltransferase EpsD | 119 | 6 | 4.2 |
| MOTHE_c21140 | transposase DDE domain protein | 1.394 | 79 | 4.2 |
| MOTHE_c08240 | bifunctional protein PyrR | 2.744 | 162 | 4.1 |
| MOTHE_c16100 | integrase core domain protein | 12.270 | 170 | 4.1 |
| MOTHE_c08250 | aspartate carbamoyltransferase | 9.336 | 247 | 4.1 |
| MOTHE_c01240 | phosphosulfolactate synthase | 267 | 16 | 4.0 |
| MOTHE_c13110 | stage II sporulation protein P (SpoIIP) | 275 | 16 | 4.0 |
| MOTHE_c10640 | threonine-phosphate decarboxylase | 6.339 | 132 | 4.0 |
| MOTHE_c01330 | CGGC domain protein | 2.107 | 137 | 3.9 |
| MOTHE_c00430 | lactate utilization protein A | 1.027 | 68 | 3.9 |
| MOTHE_c07190 | putative ATP synthase YscN | 17.529 | 229 | 3.9 |
| MOTHE_c07310 | flagellar biosynthetic protein FliP precursor | 16.023 | 75 | 3.9 |
| MOTHE_c06240 | UDP-glucose 4-epimerase | 478 | 32 | 3.9 |
| MOTHE_c04520 | SpoVR family protein | 805 | 55 | 3.9 |
| MOTHE_c07360 | flagellar biosynthesis protein FlhF | 1.763 | 121 | 3.9 |
| MOTHE_c13550 | R-phenyllactate dehydratase activator | 209 | 14 | 3.9 |
| MOTHE_c08790 | germination protease precursor | 204 | 14 | 3.9 |
| MOTHE_c13410 | spore maturation protein B | 230 | 16 | 3.8 |
| MOTHE_c13580 | spore germination protein B1 | 213 | 15 | 3.8 |
| MOTHE_c07140 | flagellar basal-body rod protein FlgC | 436 | 31 | 3.8 |
| MOTHE_c18310 | malto-oligosyltrehalose trehalohydrolase | 135 | 9 | 3.8 |
| MOTHE_c13000 | corrinoid protein CoP MtcC5 | 437 | 27 | 3.8 |
| MOTHE_c00410 | putative FAD-linked oxidoreductase | 627 | 71 | 3.8 |
| MOTHE_c19700 | hemerythrin HHE cation binding domain protein | 14.553 | 87 | 3.7 |
| MOTHE_c12520 | muramidase-2 precursor | 60 | 4 | 3.7 |
| MOTHE_c08260 | dihydroorotase | 5.052 | 384 | 3.7 |
| MOTHE_c03140 | methyltransferases MtvA1 | 118 | 9 | 3.7 |
| MOTHE_c00420 | putative FAD-linked oxidoreductase | 836 | 65 | 3.7 |
| MOTHE_c10630 | L-threonine kinase | 2.477 | 194 | 3.7 |
| MOTHE_c04960 | putative zinc metalloprotease Rip3 | 707 | 57 | 3.6 |
| MOTHE_c15870 | oxalate:formate antiporter | 98 | 8 | 3.6 |
| MOTHE_c07380 | flagellar brake protein YcgR | 737 | 62 | 3.6 |
| MOTHE_c05650 | 2-dehydro-3-deoxygluconokinase | 810 | 68 | 3.6 |
| MOTHE_c09600 | flagellar biosynthetic protein FlhB | 6 | 0 | 3.5 |
| MOTHE_c18320 | glycosyl hydrolase family 57 | 195 | 17 | 3.5 |
| MOTHE_c15860 | UDP-glucose 4-epimerase | 201 | 17 | 3.5 |
| MOTHE_c14860 | RNA polymerase sigma-F factor | 855 | 76 | 3.5 |
| MOTHE_c20600 | spore coat protein F precursor | 303 | 26 | 3.5 |
| MOTHE_c00350 | sigmaK-factor processing regulatory protein BofA | 77 | 6 | 3.5 |
| MOTHE_c14930 | stage II sporulation protein M | 1.818 | 163 | 3.5 |
| MOTHE_c18840 | 1,4-alpha-glucan branching enzyme | 6.563 | 608 | 3.4 |
| MOTHE_c13600 | stage V sporulation protein AD | 242 | 22 | 3.4 |
| MOTHE_c05280 | membrane protein of unknown function | 595 | 58 | 3.3 |
| MOTHE_c10870 | putative endonuclease 4 | 161 | 15 | 3.3 |
| MOTHE_c14880 | anti-sigma F factor antagonist | 212 | 20 | 3.3 |
| MOTHE_c00870 | stage V sporulation protein T | 1.144 | 114 | 3.3 |
| MOTHE_c08940 | sporulation integral membrane protein YlbJ | 307 | 31 | 3.3 |
| MOTHE_c10180 | D-alanyl-D-alanine carboxypeptidase DacF precursor | 15.845 | 97 | 3.3 |
| MOTHE_c10590 | cobalamin biosynthesis protein CbiB | 10.273 | 378 | 3.3 |
| MOTHE_c24800 | murein hydrolase activator NlpD precursor | 785 | 81 | 3.3 |
| MOTHE_c14870 | anti-sigma F factor | 362 | 37 | 3.3 |
| MOTHE_c18800 | 4-alpha-glucanotransferase | 4.073 | 424 | 3.3 |
| MOTHE_c25440 | elongation factor G | 31.385 | 3.366 | 3.2 |
| MOTHE_c13440 | putative spore protein YtfJ | 127 | 13 | 3.2 |
| MOTHE_c13610 | SpoVA protein | 93 | 10 | 3.2 |
| MOTHE_c15730 | sensor histidine kinase DcuS | 532 | 59 | 3.2 |
| MOTHE_c23710 | putative oxidoreductase YdhV | 154 | 16 | 3.2 |
| MOTHE_c25390 | 50S ribosomal protein L23 | 1.449 | 164 | 3.1 |
| MOTHE_c07130 | flagellar basal body rod protein FlgB | 285 | 32 | 3.1 |
| MOTHE_c22410 | hydrogenase 3 maturation protease | 57 | 6 | 3.1 |
| MOTHE_c10510 | cobalt-precorrin-2 C(20)-methyltransferase | 2.377 | 276 | 3.1 |
| MOTHE_c25370 | 30S ribosomal protein S19 | 729 | 84 | 3.1 |
| MOTHE_c25160 | 50S ribosomal protein L36 | 33 | 3 | 3.1 |
| MOTHE_c07300 | flagellar biosynthesis protein. FliO | 117 | 13 | 3.0 |
| MOTHE_c10530 | cobalamin biosynthesis protein CbiG | 3.399 | 410 | 3.0 |
| MOTHE_c25510 | 50S ribosomal protein L10 | 2.302 | 278 | 3.0 |
| MOTHE_c10550 | precorrin-6A reductase | 3.081 | 374 | 3.0 |
| MOTHE_c07210 | flagellar hook-length control protein FliK | 1.943 | 238 | 3.0 |
| MOTHE_c10580 | cobyric acid synthase | 9.241 | 947 | 3.0 |
| MOTHE_c07260 | chemotaxis protein PomA | 443 | 54 | 3.0 |
| MOTHE_c12460 | D-aminopeptidase | 130 | 16 | 3.0 |
| MOTHE_c14830 | dodecin | 24 | 2 | 3.0 |
| MOTHE_c25320 | 30S ribosomal protein S17 | 452 | 54 | 3.0 |
| MOTHE_c07640 | methylenetetrahydrofolate--tRNA-(uracil-5-)-methyltransferase TrmFO | 271 | 34 | 3.0 |
| MOTHE_c10410 | ribonuclease Y | 9.852 | 846 | 3.0 |
| MOTHE_c07100 | flagellar protein FliS | 61 | 7 | 3.0 |
| MOTHE_c18330 | maltooligosyl trehalose synthase | 178 | 22 | 3.0 |
| MOTHE_c22290 | acetyl-coenzyme A synthetase | 1.794 | 226 | 3.0 |
| MOTHE_c25310 | 50S ribosomal protein L14 | 861 | 108 | 3.0 |
| MOTHE_c07520 | heat induced stress protein YflT | 88 | 10 | 3.0 |
| MOTHE_c16250 | putative AgrB-like protein | 944 | 121 | 2.9 |
| MOTHE_c08570 | putative AgrB-like protein | 6.439 | 843 | 2.9 |
| MOTHE_c25330 | 50S ribosomal protein L29 | 297 | 38 | 2.9 |
| MOTHE_c25300 | 50S ribosomal protein L24 | 15.909 | 136 | 2.9 |
| MOTHE_c08780 | small acid-soluble spore protein C2 | 120 | 15 | 2.9 |
| MOTHE_c07220 | basal-body rod modification protein FlgD | 99 | 13 | 2.9 |
| MOTHE_c10500 | putative cobalt-precorrin-6Y C(15)-methyltransferase | 3.197 | 434 | 2.9 |
| MOTHE_c25360 | 50S ribosomal protein L22 | 2.112 | 285 | 2.9 |
| MOTHE_c07240 | flagellar basal-body rod protein FlgG | 453 | 61 | 2.9 |
| MOTHE_c21680 | sensor histidine kinase YpdA | 1.265 | 172 | 2.9 |
| MOTHE_c06930 | flagellar assembly factor FliW | 54 | 6 | 2.9 |
| MOTHE_c04950 | stage II sporulation protein Q | 15.906 | 140 | 2.9 |
| MOTHE_c07250 | flagellar protein (FlbD) | 59 | 7 | 2.9 |
| MOTHE_c25270 | 30S ribosomal protein S8 | 612 | 82 | 2.9 |
| MOTHE_c25430 | elongation factor Tu-B | 24.790 | 3.431 | 2.9 |
| MOTHE_c06330 | vancomycin B-type resistance protein VanW | 1.258 | 177 | 2.8 |
| MOTHE_c18630 | putative amidase domain protein | 306 | 43 | 2.8 |
| MOTHE_c10600 | cobyrinic acid A.C-diamide synthase | 18.789 | 722 | 2.8 |
| MOTHE_c13130 | isopentenyl-diphosphate delta-isomerase | 8.159 | 363 | 2.8 |
| MOTHE_c25420 | 30S ribosomal protein S10 | 1.522 | 217 | 2.8 |
| MOTHE_c12800 | glutamine synthetase | 155 | 22 | 2.8 |
| MOTHE_c14890 | D-alanyl-D-alanine carboxypeptidase DacF precursor | 1.770 | 261 | 2.8 |
| MOTHE_c00390 | pyruvate synthase subunit PorC | 27 | 3 | 2.8 |
| MOTHE_c24530 | amidase enhancer precursor | 349 | 51 | 2.8 |
| MOTHE_c01700 | heptaprenyl diphosphate synthase component 2 | 101 | 15 | 2.7 |
| MOTHE_c10560 | sirohydrochlorin cobaltochelatase | 1.334 | 200 | 2.7 |
| MOTHE_c10820 | BFD-like [2Fe-2S] binding domain protein | 796 | 119 | 2.7 |
| MOTHE_c25290 | 50S ribosomal protein L5 | 2.351 | 354 | 2.7 |
| MOTHE_c10620 | cobalamin synthase | 2.242 | 339 | 2.7 |
| MOTHE_c25280 | 30S ribosomal protein S14 type Z | 209 | 30 | 2.7 |
| MOTHE_c01090 | stage II sporulation protein E | 1.792 | 277 | 2.7 |
| MOTHE_c07480 | chemotaxis protein CheY | 440 | 68 | 2.7 |
| MOTHE_c23850 | NAD(P)/FAD-dependent oxidoreductase | 2.150 | 333 | 2.7 |
| MOTHE_c23900 | FAD dependent oxidoreductase | 821 | 127 | 2.7 |
| MOTHE_c00930 | spore cortex protein YabQ | 324 | 50 | 2.7 |
| MOTHE_c11730 | methyltransferase | 34.516 | 4.566 | 2.7 |
| MOTHE_c22520 | formate dehydrogenase H | 34 | 5 | 2.7 |
| MOTHE_c25340 | 50S ribosomal protein L16 | 1.866 | 291 | 2.7 |
| MOTHE_c25400 | 50S ribosomal protein L4 | 2.736 | 428 | 2.7 |
| MOTHE_c25500 | 50S ribosomal protein L7/L12 | 1.799 | 281 | 2.7 |
| MOTHE_c06890 | flagellar hook-associated protein 3 | 185 | 29 | 2.7 |
| MOTHE_c07080 | flagellar protein FlaG | 38 | 5 | 2.7 |
| MOTHE_c07470 | CheY-P phosphatase CheC | 638 | 100 | 2.7 |
| MOTHE_c25230 | 50S ribosomal protein L30 | 234 | 36 | 2.7 |
| MOTHE_c25380 | 50S ribosomal protein L2 | 16.995 | 653 | 2.6 |
| MOTHE_c25350 | 30S ribosomal protein S3 | 6.385 | 546 | 2.6 |
| MOTHE_c06290 | cyclic di-GMP phosphodiesterase response regulator RpfG | 6.268 | 550 | 2.6 |
| MOTHE_c18510 | HTH-type transcriptional activator CmpR | 580 | 96 | 2.6 |
| MOTHE_c07490 | flagellar motor switch protein FliM | 1.239 | 207 | 2.6 |
| MOTHE_c20730 | subtilisin E precursor | 78 | 12 | 2.6 |
| MOTHE_c11740 | corrinoid/iron-sulfur protein small subunit | 35.852 | 6.178 | 2.5 |
| MOTHE_c06040 | putative teichuronic acid biosynthesis glycosyltransferase TuaH | 57 | 9 | 2.5 |
| MOTHE_c10520 | cobalt-precorrin-4 C(11)-methyltransferase | 2.815 | 488 | 2.5 |
| MOTHE_c10540 | cobalt-precorrin-3B C(17)-methyltransferase | 9.467 | 564 | 2.5 |
| MOTHE_c21690 | methyltransferase MtvA3 | 7.789 | 15.930 | 2.5 |
| MOTHE_c25250 | 50S ribosomal protein L18 | 1.359 | 236 | 2.5 |
| MOTHE_c03350 | NAD-dependent malic enzyme | 3.486 | 621 | 2.5 |
| MOTHE_c11750 | maturation protein AcsF | 16.900 | 2.994 | 2.5 |
| MOTHE_c16260 | transcriptional regulatory protein YpdB | 1.812 | 324 | 2.5 |
| MOTHE_c23470 | phenylacetate-coenzyme A ligase | 5.364 | 955 | 2.5 |
| MOTHE_c24120 | transposase C of IS166 homeodomain protein | 1.006 | 179 | 2.5 |
| MOTHE_c06630 | ThiS family protein | 933 | 169 | 2.5 |
| MOTHE_c14010 | dinitrogenase iron-molybdenum cofactor | 2.536 | 452 | 2.5 |
| MOTHE_c25470 | ribosome-associated protein L7Ae-like protein | 1.625 | 295 | 2.5 |
| MOTHE_c17960 | coat F domain protein | 135 | 24 | 2.4 |
| MOTHE_c25170 | translation initiation factor IF-1 | 353 | 63 | 2.4 |
| MOTHE_c25450 | 30S ribosomal protein S7 | 7.238 | 1.326 | 2.4 |
| MOTHE_c10490 | putative cobalt-precorrin-6Y C(5)-methyltransferase | 3.428 | 632 | 2.4 |
| MOTHE_c11690 | MTHFR subunit MvhD | 14.608 | 948 | 2.4 |
| MOTHE_c19730 | glycogen synthase | 105 | 18 | 2.4 |
| MOTHE_c04150 | putative glucarate transporter | 95 | 17 | 2.4 |
| MOTHE_c14820 | ureidoglycolate lyase | 1.700 | 318 | 2.4 |
| MOTHE_c10570 | precorrin-8X methylmutase | 2.295 | 432 | 2.4 |
| MOTHE_c21040 | 2-enoate reductase FldZ | 21.121 | 548 | 2.4 |
| MOTHE_c23190 | 2.3-dimethylmalate dehydratase small subunit | 1.488 | 277 | 2.4 |
| MOTHE_c25150 | 30S ribosomal protein S13 | 1.319 | 250 | 2.4 |
| MOTHE_c08670 | ribulose-phosphate 3-epimerase | 634 | 120 | 2.4 |
| MOTHE_c21340 | protein TolB | 4.025 | 771 | 2.4 |
| MOTHE_c05140 | ribosomal silencing factor RsfS | 633 | 121 | 2.4 |
| MOTHE_c06910 | endo-1,4-beta-xylanase A precursor | 353 | 68 | 2.4 |
| MOTHE_c00920 | spore protein YabP | 166 | 31 | 2.4 |
| MOTHE_c23480 | sodium/proline symporter | 3.265 | 632 | 2.4 |
| MOTHE_c10670 | EamA-like transporter family protein | 104 | 20 | 2.3 |
| MOTHE_c14770 | methyl-accepting chemotaxis protein McpA | 543 | 106 | 2.3 |
| MOTHE_c21630 | branched-chain-amino-acid aminotransferase | 344 | 66 | 2.3 |
| MOTHE_c25460 | 30S ribosomal protein S12 | 7.579 | 1.500 | 2.3 |
| MOTHE_c16090 | transposase | 286 | 55 | 2.3 |
| MOTHE_c18120 | acyl-coenzyme A thioesterase PaaI | 410 | 82 | 2.3 |
| MOTHE_c23430 | HTH-type transcriptional regulator LutR | 708 | 142 | 2.3 |
| MOTHE_c13380 | ribosomal large subunit pseudouridine synthase B | 334 | 67 | 2.3 |
| MOTHE_c25410 | 50S ribosomal protein L3 | 17.794 | 764 | 2.3 |
| MOTHE_c05120 | RNA recognition motif protein | 3.306 | 676 | 2.3 |
| MOTHE_c09570 | enterobactin exporter EntS | 156 | 31 | 2.3 |
| MOTHE_c23210 | 2,3-dimethylmalate dehydratase large subunit | 914 | 185 | 2.3 |
| MOTHE_c10480 | cobalt-precorrin-6A synthase | 7.354 | 1.505 | 2.3 |
| MOTHE_c14490 | spore germination protein YndE | 22 | 3 | 2.3 |
| MOTHE_c10130 | riboflavin biosynthesis protein RibF | 1.021 | 209 | 2.3 |
| MOTHE_c24110 | transposase IS66 family protein | 6.385 | 1.319 | 2.3 |
| MOTHE_c14990 | arginine repressor | 253 | 52 | 2.3 |
| MOTHE_c00330 | recombination protein RecR | 758 | 159 | 2.3 |
| MOTHE_c11670 | MTHFR subunit MetF | 15.775 | 26.440 | 2.3 |
| MOTHE_c13420 | spore maturation protein A | 231 | 47 | 2.3 |
| MOTHE_c21700 | corrinoid protein MtvC5 | 4.994 | 1.044 | 2.3 |
| MOTHE_c23970 | macrolide export protein MacA | 485 | 100 | 2.3 |
| MOTHE_c10080 | putative ribosomal protein YlxQ | 253 | 53 | 2.2 |
| MOTHE_c25260 | 50S ribosomal protein L6 | 15.242 | 453 | 2.2 |
| MOTHE_c10100 | ribosome-binding factor A | 288 | 59 | 2.2 |
| MOTHE_c12170 | Low-affinity inorganic phosphate transporter 1 | 68 | 14 | 2.2 |
| MOTHE_c15880 | oxalate:formate antiporter | 476 | 103 | 2.2 |
| MOTHE_c23250 | acetolactate synthase large subunit | 7.529 | 1.635 | 2.2 |
| MOTHE_c10610 | nicotinate-nucleotide--dimethylbenzimidazole phosphoribosyltransferase | 22.314 | 823 | 2.2 |
| MOTHE_c07090 | flagellar hook-associated protein 2 | 434 | 95 | 2.2 |
| MOTHE_c07500 | flagellar motor switch protein FliN | 1.209 | 266 | 2.2 |
| MOTHE_c13830 | RNA polymerase-associated protein RapA | 51 | 10 | 2.2 |
| MOTHE_c15000 | NAD kinase | 1.717 | 380 | 2.2 |
| MOTHE_c23220 | 2-isopropylmalate synthase | 4.250 | 15.865 | 2.2 |
| MOTHE_c25240 | 30S ribosomal protein S5 | 2.236 | 497 | 2.2 |
| MOTHE_c16270 | sensor histidine kinase DcuS | 1.039 | 235 | 2.1 |
| MOTHE_c18340 | trehalose synthase | 88 | 19 | 2.1 |
| MOTHE_c25490 | DNA-directed RNA polymerase subunit beta | 26.500 | 27.624 | 2.1 |
| MOTHE_c10470 | bifunctional adenosylcobalamin biosynthesis protein CobU | 1.996 | 460 | 2.1 |
| MOTHE_c05130 | putative nicotinate-nucleotide adenylyltransferase | 1.619 | 378 | 2.1 |
| MOTHE_c08290 | orotidine 5'-phosphate decarboxylase | 883 | 205 | 2.1 |
| MOTHE_c05160 | citrate transporter | 1.755 | 412 | 2.1 |
| MOTHE_c06250 | PIN domain protein | 245 | 57 | 2.1 |
| MOTHE_c09430 | NAD(P)H-quinone oxidoreductase chain 4 1 | 4.553 | 1.067 | 2.1 |
| MOTHE_c22370 | hydrogenase isoenzymes formation protein HypC | 4 | 0 | 2.1 |
| MOTHE_c00140 | phosphocarrier protein HPr | 53 | 11 | 2.1 |
| MOTHE_c22530 | formate dehydrogenase H | 4 | 0 | 2.1 |
| MOTHE_c11770 | corrinoid/iron-sulfur protein large subunit CoFeSP (WLP) | 47.619 | 12.684 | 2.1 |
| MOTHE_c22430 | formate hydrogenlyase subunit 7 | 115 | 27 | 2.1 |
| MOTHE_c23980 | putative multidrug resistance protein EmrK | 283 | 67 | 2.1 |
| MOTHE_c09050 | acyl carrier protein | 656 | 155 | 2.1 |
| MOTHE_c23240 | acetolactate synthase small subunit | 1.593 | 384 | 2.1 |
| MOTHE_c11780 | carbon monoxide dehydrogenase/acetyl-CoA synthase subunit alpha | 54.317 | 13.372 | 2.0 |
| MOTHE_c22450 | formate hydrogenlyase subunit 5 precursor | 180 | 42 | 2.0 |
| MOTHE_c22750 | ArsR family metal-binding transcriptional regulator | 29 | 6 | 2.0 |
| MOTHE_c19530 | 1.4-alpha-glucan branching enzyme | 66 | 15 | 2.0 |
| MOTHE_c11700 | MTHFR subunit HdrA | 73.761 | 18.224 | 2.0 |
| MOTHE_c01690 | tetraprenyl-beta-curcumene synthase | 147 | 36 | 2.0 |
| MOTHE_c08660 | putative ribosome biogenesis GTPase RsgA | 1.251 | 308 | 2.0 |
| MOTHE_c11710 | MTHFR subunit HdrB | 16.070 | 4.018 | 2.0 |
| MOTHE_c13770 | CotJB protein | 8 | 1 | 2.0 |
| MOTHE_c13320 | universal stress protein family protein | 100 | 24 | 2.0 |
| MOTHE_c11800 | septum site-determining protein MinD | 6.486 | 23.826 | 2.0 |
| MOTHE_c13330 | Trk system potassium uptake protein TrkA | 88 | 21 | 2.0 |
| MOTHE_c23490 | potassium/proton antiporter | 13 | 2 | 2.0 |
| MOTHE_c02120 | sporulation protein YpeB | 115 | 28 | 2.0 |
| MOTHE_c13340 | Trk system potassium uptake protein TrkA | 192 | 48 | 2.0 |
| MOTHE_c15010 | 16S/23S rRNA (cytidine-2'-O)-methyltransferase TlyA | 1.299 | 331 | 2.0 |
| MOTHE_c22610 | YKOF-related family protein | 55 | 13 | 2.0 |
| MOTHE_c23440 | pyrimidine-nucleoside phosphorylase | 2.110 | 544 | 2.0 |

**Supplementary table 2: The most downregulated genes of *M. thermoacetica* during growth on methanol.**

| **gene** | **annotation** | **substrate** | | **Log_2_ (fold change)** |
| --- | --- | --- | --- | --- |
|  |  | **methanol** | **glucose** |  |
| MOTHE_c24170 | Copper amine oxidase-like protein | 0 | 74.558 | -21.3 |
| MOTHE_c18140 | putative prophage phiRv2 integrase | 0 | 1.140 | -14.5 |
| MOTHE_c18240 | transcription elongation factor Elf1 | 0 | 16.050 | -14.5 |
| MOTHE_c24160 | outer membrane efflux protein | 0 | 856 | -14.2 |
| MOTHE_c24080 | tRNA(fMet)-specific endonuclease VapC | 0 | 672 | -13.8 |
| MOTHE_c24150 | endo-1,4-beta-xylanase A precursor | 0 | 384 | -13.0 |
| MOTHE_c24090 | antitoxin YefM | 0 | 370 | -12.9 |
| MOTHE_c24270 | carbohydrate diacid regulator | 0 | 324 | -12.7 |
| MOTHE_c18160 | type II toxin-antitoxin system MqsA family antitoxin | 0 | 178 | -11.4 |
| MOTHE_c16220 | transposase | 0 | 157 | -11.3 |
| MOTHE_c18150 | predicted RNase H-like HicB family nuclease | 0 | 129 | -10.9 |
| MOTHE_c18170 | putative transcriptional regulator | 0 | 120 | -10.9 |
| MOTHE_c18900 | MarR family protein | 0 | 510 | -10.8 |
| MOTHE_c12840 | 8-oxoguanine deaminase | 0 | 79 | -10.3 |
| MOTHE_c11280 | alkaline phosphatase precursor | 125 | 150.353 | -10.2 |
| MOTHE_c18520 | transposase | 1 | 1.678 | -10.0 |
| MOTHE_c24240 | HAAS domain-containing protein | 0 | 63 | -9.9 |
| MOTHE_c18910 | putative transporter | 0 | 50 | -9.6 |
| MOTHE_c24200 | putative sulfoacetate transporter SauU | 0 | 40 | -9.2 |
| MOTHE_c18280 | DNA primase | 0 | 40 | -9.0 |
| MOTHE_c24190 | uroporphyrinogen decarboxylase | 0 | 28 | -8.6 |
| MOTHE_c24230 | lineage-specific thermal regulator protein | 0 | 28 | -8.3 |
| MOTHE_c24210 | uroporphyrinogen decarboxylase | 0 | 22 | -8.1 |
| MOTHE_c12850 | 8-oxoguanine deaminase | 1 | 209 | -8.1 |
| MOTHE_c20120 | O-acetylserine sulfhydrylase | 118 | 21.177 | -7.5 |
| MOTHE_c11590 | FeoA domain protein | 74 | 11.943 | -7.3 |
| MOTHE_c09750 | plasmid stabilization system protein | 0 | 17 | -7.3 |
| MOTHE_c14410 | succinate dehydrogenase/fumarate reductase iron-sulfur subunit | 156 | 24.533 | -7.3 |
| MOTHE_c14240 | ferrous iron transport protein A | 10 | 1.501 | -7.2 |
| MOTHE_c04110 | phosphate-binding protein PstS 1 precursor | 491 | 69.602 | -7.1 |
| MOTHE_c14230 | FeoA domain protein | 17 | 2.124 | -7.0 |
| MOTHE_c14420 | heterodisulfide reductase subunit A2 | 790 | 93.605 | -6.9 |
| MOTHE_c14430 | nitric oxide reductase FlRd-NAD^+^ reductase | 321 | 31.887 | -6.6 |
| MOTHE_c14220 | ferrous iron transport protein B | 422 | 39.880 | -6.5 |
| MOTHE_c15680 | primary amine oxidase precursor | 2 | 169 | -6.5 |
| MOTHE_c20130 | carbon monoxide dehydrogenase 1 | 313 | 28.545 | -6.5 |
| MOTHE_c04090 | Hsp20/alpha crystallin family protein | 44 | 3.902 | -6.5 |
| MOTHE_c11580 | ferrous iron transport protein B | 1.074 | 90.315 | -6.4 |
| MOTHE_c13870 | fructoselysine 3-epimerase | 38 | 3.015 | -6.3 |
| MOTHE_c03550 | 2-dehydro-3-deoxygluconokinase | 236 | 16.083 | -6.1 |
| MOTHE_c03560 | KHG/KDPG aldolase | 169 | 11.261 | -6.1 |
| MOTHE_c20150 | NitT/TauT family transport system permease protein | 54 | 17.439 | -5.9 |
| MOTHE_c18540 | L-lactate dehydrogenase | 63 | 3.400 | -5.8 |
| MOTHE_c18360 | YmaF family protein | 0 | 7 | -5.5 |
| MOTHE_c02190 | glutamate racemase | 33 | 1.498 | -5.5 |
| MOTHE_c20140 | putative aliphatic sulfonates-binding protein precursor | 116 | 5.187 | -5.5 |
| MOTHE_c03540 | K^+^-stimulated pyrophosphate-energized sodium pump | 448 | 17.360 | -5.3 |
| MOTHE_c20160 | aliphatic sulfonates import ATP-binding protein SsuB | 107 | 4.058 | -5.2 |
| MOTHE_c14740 | sn-glycerol-3-phosphate-binding periplasmic protein UgpB precursor | 126 | 11.198 | -5.2 |
| MOTHE_c17280 | electron bifurcating hydrogenase subunit HydC | 54 | 1.834 | -5.1 |
| MOTHE_c15770 | flagellar assembly protein H | 13 | 497 | -5.1 |
| MOTHE_c02530 | HTH-type transcriptional regulator ImmR | 6 | 191 | -5.0 |
| MOTHE_c16600 | HTH-type transcriptional regulator CymR | 555 | 16.464 | -4.9 |
| MOTHE_c17260 | electron bifurcating hydrogenase subunit HydA | 414 | 15.673 | -4.9 |
| MOTHE_c17270 | electron bifurcating hydrogenase subunit HydB | 437 | 12.637 | -4.9 |
| MOTHE_c23070 | N-6 DNA methylase | 46 | 1.298 | -4.8 |
| MOTHE_c22300 | sensor histidine kinase YehU | 128 | 3.410 | -4.7 |
| MOTHE_c23820 | anaerobic glycerol-3-phosphate dehydrogenase subunit C | 4.473 | 29.467 | -4.7 |
| MOTHE_c14730 | sn-glycerol-3-phosphate transport system permease protein UgpA | 40 | 1.091 | -4.7 |
| MOTHE_c19460 | perchlorate reductase subunit alpha precursor | 126 | 3.149 | -4.6 |
| MOTHE_c14210 | cyclic di-GMP phosphodiesterase response regulator RpfG | 261 | 6.476 | -4.6 |
| MOTHE_c16590 | cysteine desulfurase IscS | 4.320 | 110.073 | -4.6 |
| MOTHE_c19270 | hydroperoxide reductase | 1.093 | 24.725 | -4.6 |
| MOTHE_c23830 | putative FAD-linked oxidoreductase | 1.797 | 42.196 | -4.6 |
| MOTHE_c20110 | putative adenylyltransferase/sulfurtransferase MoeZ | 1.590 | 36.988 | -4.5 |
| MOTHE_c02200 | OPT oligopeptide transporter protein | 151 | 3.527 | -4.5 |
| MOTHE_c14710 | sn-glycerol-3-phosphate import ATP-binding protein UgpC | 71 | 1.648 | -4.5 |
| MOTHE_c20250 | 2-oxoglutarate ferredoxin oxidoreductase subunit delta | 0 | 5 | -4.5 |
| MOTHE_c06670 | 4-hydroxythreonine-4-phosphate dehydrogenase 2 | 54 | 1.229 | -4.5 |
| MOTHE_c08870 | peroxide-responsive repressor PerR | 168 | 3.740 | -4.5 |
| MOTHE_c19450 | tetrathionate reductase subunit B precursor | 21 | 471 | -4.5 |
| MOTHE_c11560 | phosphate propanoyltransferase | 850 | 18.699 | -4.5 |
| MOTHE_c19440 | putative hydrogenase 2 b cytochrome subunit | 51 | 1.125 | -4.5 |
| MOTHE_c14720 | L-arabinose transport system permease protein AraQ | 29 | 622 | -4.4 |
| MOTHE_c20310 | cystathionine gamma-lyase | 31 | 642 | -4.4 |
| MOTHE_c20100 | anaerobic sulfite reductase subunit C | 780 | 15.464 | -4.3 |
| MOTHE_c03840 | HTH-type transcriptional activator CmpR | 197 | 3.808 | -4.3 |
| MOTHE_c05800 | HTH-type transcriptional repressor PurR | 31 | 601 | -4.3 |
| MOTHE_c16580 | iron-sulfur cluster assembly scaffold protein IscU | 775 | 14.542 | -4.2 |
| MOTHE_c22310 | sensory transduction protein LytR | 62 | 1.182 | -4.2 |
| MOTHE_c05880 | IS2 transposase TnpB | 245 | 4.577 | -4.2 |
| MOTHE_c17130 | cysteine synthase | 577 | 10.683 | -4.2 |
| MOTHE_c23810 | lactate utilization protein C | 462 | 8.281 | -4.2 |
| MOTHE_c06680 | Gnt-II system L-idonate transporter | 38 | 647 | -4.1 |
| MOTHE_c23790 | HTH-type transcriptional regulator LutR | 559 | 21.707 | -4.1 |
| MOTHE_c03160 | integrase core domain protein | 430 | 21.549 | -4.1 |
| MOTHE_c02250 | 2,5-dihydroxypyridine 5,6-dioxygenase | 129 | 2.117 | -4.0 |
| MOTHE_c20050 | sensor histidine kinase YehU | 522 | 20.609 | -4.0 |
| MOTHE_c01870 | putative transposase. YhgA-like | 104 | 6.861 | -4.0 |
| MOTHE_c08850 | hemin import ATP-binding protein HmuV | 461 | 7.216 | -4.0 |
| MOTHE_c21490 | cytochrome bd ubiquinol oxidase subunit 1 | 124 | 2.897 | -4.0 |
| MOTHE_c20080 | sulfur carrier protein ThiS | 75 | 1.134 | -3.9 |
| MOTHE_c14040 | serine dehydratase alpha chain | 27 | 403 | -3.9 |
| MOTHE_c02410 | putative sigma-54 modulation protein | 588 | 8.716 | -3.9 |
| MOTHE_c19840 | aminomethyltransferase | 298 | 4.364 | -3.9 |
| MOTHE_c03530 | transcriptional regulator KdgR | 188 | 10.935 | -3.8 |
| MOTHE_c08860 | high-affinity zinc uptake system binding-protein ZnuA precursor | 702 | 10.023 | -3.8 |
| MOTHE_c08840 | manganese transport system membrane protein MntB | 105 | 1.490 | -3.8 |
| MOTHE_c23800 | lactate utilization protein B | 1.992 | 27.617 | -3.8 |
| MOTHE_c22010 | cellulosome-anchoring protein precursor | 231 | 12.290 | -3.8 |
| MOTHE_c21190 | PIN domain protein | 110 | 5.761 | -3.7 |
| MOTHE_c03570 | N-acetylglucosamine repressor | 5 | 63 | -3.7 |
| MOTHE_c22670 | antitoxin VapB47 | 25 | 336 | -3.7 |
| MOTHE_c11540 | transcriptional regulator KdgR | 120 | 6.183 | -3.7 |
| MOTHE_c21540 | methyltransferases MtaA | 147 | 1.894 | -3.7 |
| MOTHE_c12580 | tRNA modification GTPase MnmE | 503 | 11.513 | -3.7 |
| MOTHE_c02000 | endo-1,4-beta-xylanase A precursor | 967 | 11.935 | -3.6 |
| MOTHE_c13510 | spore coat protein SA | 1.027 | 12.364 | -3.6 |
| MOTHE_c17440 | CRISPR associated protein Cas6 | 19 | 227 | -3.6 |
| MOTHE_c12570 | flagellin N-methylase | 191 | 2.257 | -3.6 |
| MOTHE_c14400 | HTH-type transcriptional regulator GlnR | 7 | 85 | -3.6 |
| MOTHE_c21360 | transcriptional repressor SdpR | 50 | 662 | -3.5 |
| MOTHE_c06370 | glucitol operon repressor | 71 | 830 | -3.5 |
| MOTHE_c21370 | immunity protein SdpI | 143 | 15.532 | -3.5 |
| MOTHE_c20320 | cystathionine beta-lyase MetC | 76 | 857 | -3.5 |
| MOTHE_c14200 | rhodocoxin reductase | 844 | 9.475 | -3.5 |
| MOTHE_c02450 | coenzyme PQQ synthesis protein E | 1.886 | 16.842 | -3.4 |
| MOTHE_c15940 | HTH-type transcriptional regulator McbR | 107 | 15.130 | -3.4 |
| MOTHE_c06280 | ribonuclease VapC20 | 22 | 244 | -3.4 |
| MOTHE_c01990 | endo-1,4-beta-xylanase A precursor | 2.288 | 23.973 | -3.4 |
| MOTHE_c02790 | phosphoglycerate kinase | 3.616 | 36.464 | -3.3 |
| MOTHE_c01710 | transcriptional regulator CtsR | 216 | 2.130 | -3.3 |
| MOTHE_c03150 | chromosomal replication initiator protein DnaA | 143 | 1.407 | -3.3 |
| MOTHE_c17250 | arylesterase precursor | 295 | 17.120 | -3.3 |
| MOTHE_c19130 | reverse rubrerythrin-1 | 4.596 | 59.998 | -3.3 |
| MOTHE_c14290 | ATP-dependent (S)-NAD(P)H-hydrate dehydratase | 1.170 | 11.229 | -3.3 |
| MOTHE_c03210 | transposase | 102 | 968 | -3.2 |
| MOTHE_c16390 | NAD(P)H-quinone oxidoreductase subunit I. chloroplastic | 565 | 11.203 | -3.2 |
| MOTHE_c04450 | nitrite reductase [NAD(P)H] | 424 | 13.790 | -3.2 |
| MOTHE_c23780 | glycolate permease GlcA | 6.044 | 14.194 | -3.2 |
| MOTHE_c20040 | sensory transduction protein LytR | 173 | 1.614 | -3.2 |
| MOTHE_c11480 | demethylrebeccamycin-D-glucose O-methyltransferase | 485 | 4.424 | -3.2 |
| MOTHE_c04430 | cyclic di-GMP phosphodiesterase response regulator RpfG | 1.130 | 10.133 | -3.2 |
| MOTHE_c02800 | triosephosphate isomerase | 2.813 | 25.147 | -3.2 |
| MOTHE_c06720 | PemK-like protein | 100 | 15.821 | -3.2 |
| MOTHE_c20850 | ATP phosphoribosyltransferase regulatory subunit | 2.293 | 20.646 | -3.2 |
| MOTHE_c21250 | HTH-type transcriptional activator TipA | 34 | 305 | -3.1 |
| MOTHE_c02810 | 2,3-bisphosphoglycerate-independent phosphoglycerate mutase | 5.848 | 51.280 | -3.1 |
| MOTHE_c20840 | ATP phosphoribosyltransferase | 888 | 7.814 | -3.1 |
| MOTHE_c21380 | (R)-stereoselective amidase | 398 | 3.425 | -3.1 |
| MOTHE_c22870 | transcriptional regulator PadR-like family protein | 125 | 15.995 | -3.1 |
| MOTHE_c24070 | HTH-type transcriptional repressor NicS | 62 | 539 | -3.1 |
| MOTHE_c21500 | cytochrome bd-I ubiquinol oxidase subunit 2 | 88 | 1.154 | -3.1 |
| MOTHE_c14190 | ribonuclease BN | 503 | 14.301 | -3.0 |
| MOTHE_c08880 | putative competence-damage inducible protein | 1.530 | 12.263 | -3.0 |
| MOTHE_c16380 | sulfite reductase. dissimilatory-type subunit gamma | 1.023 | 8.002 | -3.0 |
| MOTHE_c16360 | sulfite reductase. dissimilatory-type subunit alpha | 1.357 | 10.489 | -3.0 |
| MOTHE_c04720 | putative fluoride ion transporter CrcB | 566 | 18.275 | -2.9 |
| MOTHE_c16340 | cobyrinic acid A.C-diamide synthase | 15.856 | 7.256 | -2.9 |
| MOTHE_c07000 | N,N'-diacetyllegionaminic acid synthase | 11 | 84 | -2.9 |
| MOTHE_c08020 | laccase domain protein | 2.358 | 17.594 | -2.9 |
| MOTHE_c25760 | putative phosphatase YcdX | 401 | 2.967 | -2.9 |
| MOTHE_c21550 | carbohydrate diacid regulator | 73 | 542 | -2.9 |
| MOTHE_c20220 | putative sulfoacetate--CoA ligase | 31 | 235 | -2.9 |
| MOTHE_c16560 | tRNA-specific 2-thiouridylase MnmA | 1.627 | 11.582 | -2.8 |
| MOTHE_c22110 | putative transposase | 763 | 17.750 | -2.8 |
| MOTHE_c03040 | transcriptional regulator KdgR | 7 | 52 | -2.8 |
| MOTHE_c12040 | nicotinate dehydrogenase large molybdopterin subunit | 205 | 6.187 | -2.8 |
| MOTHE_c13800 | nucleotidyltransferase domain protein | 319 | 2.226 | -2.8 |
| MOTHE_c22660 | diflavin flavoprotein A 1 | 1.267 | 8.851 | -2.8 |
| MOTHE_c12830 | nitrogen regulatory protein P-II | 9 | 62 | -2.8 |
| MOTHE_c01190 | phosphate transport system permease protein PstA | 448 | 2.933 | -2.7 |
| MOTHE_c16350 | sulfite reductase. dissimilatory-type subunit beta | 1.275 | 8.264 | -2.7 |
| MOTHE_c21220 | putative transposase. YhgA-like | 661 | 6.378 | -2.7 |
| MOTHE_c14130 | putative ABC transporter ATP-binding protein | 3 | 21 | -2.7 |
| MOTHE_c06750 | putative competence-damage inducible protein | 903 | 11.726 | -2.6 |
| MOTHE_c12680 | desulfoferrodoxin | 904 | 6.516 | -2.6 |
| MOTHE_c05480 | cytidine deaminase | 820 | 17.518 | -2.6 |
| MOTHE_c10450 | DNA polymerase III PolC-type | 233 | 1.439 | -2.6 |
| MOTHE_c14340 | periplasmic solute binding protein family protein | 105 | 646 | -2.6 |
| MOTHE_c18580 | thiol:disulfide interchange protein DsbD | 42 | 263 | -2.6 |
| MOTHE_c01560 | long-chain acyl-[acyl-carrier-protein] reductase | 300 | 1.827 | -2.6 |
| MOTHE_c11350 | HTH-type transcriptional repressor YtrA | 58 | 358 | -2.6 |
| MOTHE_c23660 | 2-aminophenol 1.6-dioxygenase alpha subunit | 1.298 | 20.492 | -2.6 |
| MOTHE_c16370 | sulfurtransferase TusE | 169 | 1.025 | -2.6 |
| MOTHE_c25570 | putative 4-amino-4-deoxy-L-arabinose-phosphoundecaprenol flippase subunit ArnE | 15 | 98 | -2.6 |
| MOTHE_c02580 | cellulosome-anchoring protein precursor | 3 | 23 | -2.6 |
| MOTHE_c01180 | phosphate transport system permease protein PstC | 532 | 3.193 | -2.6 |
| MOTHE_c18000 | coenzyme PQQ synthesis protein E | 499 | 2.953 | -2.6 |
| MOTHE_c16510 | tRNA/tmRNA (uracil-C(5))-methyltransferase | 609 | 3.588 | -2.6 |
| MOTHE_c15440 | type II secretion system protein F | 130 | 753 | -2.5 |
| MOTHE_c13790 | HEPN domain protein | 342 | 5.463 | -2.5 |
| MOTHE_c18790 | EamA-like transporter family protein | 54 | 311 | -2.5 |
| MOTHE_c20820 | imidazoleglycerol-phosphate dehydratase | 914 | 5.199 | -2.5 |
| MOTHE_c04780 | Lon protease 2 | 445 | 8.360 | -2.5 |
| MOTHE_c12670 | high molecular weight rubredoxin | 105 | 744 | -2.5 |
| MOTHE_c00650 | ferredoxin-like protein FixX | 3 | 19 | -2.5 |
| MOTHE_c00740 | alkaline phosphatase synthesis transcriptional regulatory protein PhoP | 1.144 | 6.400 | -2.5 |
| MOTHE_c00620 | electron transfer flavoprotein-ubiquinone oxidoreductase EtfB | 175 | 975 | -2.5 |
| MOTHE_c15460 | type II secretion system protein E | 266 | 1.460 | -2.5 |
| MOTHE_c02520 | putative transposase | 315 | 1.712 | -2.4 |
| MOTHE_c02820 | enolase | 5.311 | 28.808 | -2.4 |
| MOTHE_c12020 | vanillate:cobalamin methyltransferase MtvB2 | 171 | 933 | -2.4 |
| MOTHE_c17120 | cysteine-rich secretory protein family protein | 313 | 1.695 | -2.4 |
| MOTHE_c21480 | HTH-type transcriptional regulator CymR | 106 | 931 | -2.4 |
| MOTHE_c02660 | UvrABC system protein B | 1.167 | 27.002 | -2.4 |
| MOTHE_c17600 | agmatinase | 328 | 1.753 | -2.4 |
| MOTHE_c01750 | dTDP-glucose 4,6-dehydratase | 12 | 66 | -2.4 |
| MOTHE_c13750 | chaperone DmsD | 14 | 80 | -2.4 |
| MOTHE_c14060 | iron-sulfur cluster assembly scaffold protein IscU | 103 | 544 | -2.4 |
| MOTHE_c20800 | 1-(5-phosphoribosyl)-5-[(5-phosphoribosylamino)methylideneamino] imidazole-4-carboxamide isomerase | 802 | 29.736 | -2.4 |
| MOTHE_c01930 | putative transposase, YhgA-like | 393 | 2.028 | -2.4 |
| MOTHE_c21510 | ATP-binding/permease protein CydD | 154 | 1.273 | -2.3 |
| MOTHE_c00660 | bacterioferritin | 16 | 87 | -2.3 |
| MOTHE_c18930 | nickel import ATP-binding protein NikO | 520 | 8.722 | -2.3 |
| MOTHE_c15370 | competence protein A | 145 | 732 | -2.3 |
| MOTHE_c17820 | ribonuclease VapC20 | 74 | 380 | -2.3 |
| MOTHE_c20780 | phosphoribosyl-ATP pyrophosphatase | 973 | 4.886 | -2.3 |
| MOTHE_c20830 | histidinol dehydrogenase | 2.194 | 10.972 | -2.3 |
| MOTHE_c12030 | nicotinate dehydrogenase medium molybdopterin subunit | 126 | 624 | -2.3 |
| MOTHE_c18950 | fused nickel transport protein NikMN | 13.173 | 21.260 | -2.3 |
| MOTHE_c09470 | acetophenone carboxylase alpha subunit | 12.472 | 9.949 | -2.3 |
| MOTHE_c06420 | methylthioribose-1-phosphate isomerase | 10.791 | 8.587 | -2.3 |
| MOTHE_c00640 | electron transfer flavoprotein-ubiquinone oxidoreductase EtfC | 168 | 817 | -2.3 |
| MOTHE_c01200 | phosphate import ATP-binding protein PstB 3 | 269 | 1.285 | -2.3 |
| MOTHE_c19810 | putative glycine dehydrogenase (decarboxylating) subunit 2 | 4.174 | 19.821 | -2.3 |
| MOTHE_c02560 | bacterial Ig-like domain (group 2) | 11 | 56 | -2.2 |
| MOTHE_c24910 | putative beta-barrel protein YwiB | 283 | 15.442 | -2.2 |
| MOTHE_c19510 | CAAX amino terminal protease self- immunity | 370 | 16.481 | -2.2 |
| MOTHE_c00630 | electron transfer flavoprotein-ubiquinone oxidoreductase EtfA | 168 | 788 | -2.2 |
| MOTHE_c01720 | UvrB/uvrC motif protein | 484 | 2.251 | -2.2 |
| MOTHE_c06730 | IMPACT family member YigZ | 153 | 714 | -2.2 |
| MOTHE_c21400 | surface layer protein precursor | 18.115 | 19.407 | -2.2 |
| MOTHE_c22120 | poly-beta-1.6-N-acetyl-D-glucosamine synthase | 46 | 216 | -2.2 |
| MOTHE_c15420 | type 4 prepilin-like proteins leader peptide-processing enzyme | 43 | 199 | -2.2 |
| MOTHE_c24540 | UDP-N-acetylglucosamine 1-carboxyvinyltransferase 1 | 1.444 | 6.570 | -2.2 |
| MOTHE_c02170 | zinc ribbon domain protein | 33 | 155 | -2.2 |
| MOTHE_c05840 | phosphoenolpyruvate carboxykinase | 42 | 197 | -2.2 |
| MOTHE_c20810 | imidazole glycerol phosphate synthase subunit HisH 1 | 621 | 17.157 | -2.2 |
| MOTHE_c03680 | biofilm growth-associated repressor | 4 | 24 | -2.2 |
| MOTHE_c00100 | glucitol operon repressor | 80 | 359 | -2.2 |
| MOTHE_c01740 | negative regulator of genetic competence ClpC/MecB | 5.979 | 26.364 | -2.1 |
| MOTHE_c12350 | hydroxyacylglutathione hydrolase | 150 | 663 | -2.1 |
| MOTHE_c12380 | hexuronate transporter | 98 | 433 | -2.1 |
| MOTHE_c19820 | putative glycine dehydrogenase (decarboxylating) subunit 1 | 3.926 | 17.266 | -2.1 |
| MOTHE_c20790 | imidazole glycerol phosphate synthase subunit HisF | 1.079 | 4.751 | -2.1 |
| MOTHE_c23650 | cyclic pyranopterin monophosphate synthase | 857 | 3.767 | -2.1 |
| MOTHE_c14080 | R-phenyllactate dehydratase beta subunit | 4.204 | 18.391 | -2.1 |
| MOTHE_c21030 | cell wall-binding protein YocH precursor | 31 | 141 | -2.1 |
| MOTHE_c02050 | periplasmic protein | 74 | 328 | -2.1 |
| MOTHE_c06760 | putative serine protease HhoB precursor | 916 | 3.970 | -2.1 |
| MOTHE_c06430 | L-fuculose phosphate aldolase | 906 | 3.869 | -2.1 |
| MOTHE_c07040 | flagellin C | 134 | 574 | -2.1 |
| MOTHE_c14140 | putative ABC transporter permease protein | 11 | 53 | -2.1 |
| MOTHE_c01730 | putative ATP:guanido phosphotransferase | 1.507 | 6.352 | -2.1 |
| MOTHE_c01860 | DNA replication and repair protein RecF | 15 | 69 | -2.1 |
| MOTHE_c04480 | glucose-6-phosphate isomerase | 2.010 | 10.612 | -2.1 |
| MOTHE_c05690 | ribonuclease H | 274 | 1.145 | -2.1 |
| MOTHE_c12220 | inner membrane protein YgaZ | 277 | 1.159 | -2.1 |
| MOTHE_c13740 | DMSO reductase subunit DmsA3 | 133 | 556 | -2.1 |
| MOTHE_c18870 | neopullulanase 2 | 446 | 1.860 | -2.1 |
| MOTHE_c18420 | PhoU domain protein | 5 | 25 | -2.0 |
| MOTHE_c19310 | carboxymuconolactone decarboxylase family protein | 1.108 | 25.807 | -2.0 |
| MOTHE_c20770 | methyl-accepting chemotaxis protein McpB | 2.466 | 21.864 | -2.0 |
| MOTHE_c02780 | glyceraldehyde-3-phosphate dehydrogenase | 21.679 | 39.794 | -2.0 |
| MOTHE_c05980 | cellulosome-anchoring protein precursor | 10.817 | 7.261 | -2.0 |
| MOTHE_c14110 | O-methyltransferase | 3 | 16 | -2.0 |
| MOTHE_c14370 | methyltransferases corrinoid protein complex MtaAC | 28.551 | 15.290 | -2.0 |
| MOTHE_c22100 | transcriptional activator protein CopR | 64 | 266 | -2.0 |
| MOTHE_c02650 | YcfA-like protein | 27 | 117 | -2.0 |
| MOTHE_c24960 | ribosomal RNA large subunit methyltransferase H | 120 | 484 | -2.0 |
| MOTHE_c16330 | HTH-type transcriptional regulator SinR | 483 | 1.930 | -2.0 |
| MOTHE_c23640 | 2.5-dihydroxypyridine 5.6-dioxygenase | 795 | 3.175 | -2.0 |
| MOTHE_c17360 | type IV secretory system conjugative DNA transfer | 1 | 7 | -2.0 |
| MOTHE_c12630 | S-adenosylmethionine decarboxylase proenzyme precursor | 1.252 | 4.948 | -2.0 |
| MOTHE_c14330 | manganese transport system membrane protein MntB | 30 | 122 | -2.0 |
| MOTHE_c18940 | nickel transport protein NikQ | 1.363 | 5.320 | -2.0 |
| MOTHE_c03980 | imidazolonepropionase | 368 | 7.139 | -2.0 |
| MOTHE_c13270 | anthranilate synthase component 1 | 199 | 778 | -2.0 |
| MOTHE_c23300 | zinc ribbon domain protein | 28 | 112 | -2.0 |

**Supplementary table 3: The most upregulated genes of *M. thermoacetica* during growth on methanol + DMSO.**

| **gene** | **annotation** | **substrate** | | **Log_2_ (fold change)** |
| --- | --- | --- | --- | --- |
|  |  | **methanol + DMSO** | **methanol** |  |
| MOTHE_c14970 | SpoIVB peptidase precursor | 18.050 | 15 | 8.0 |
| MOTHE_c08780 | small acid-soluble spore protein C2 | 23.107 | 143 | 7.3 |
| MOTHE_c12520 | muramidase-2 precursor | 20.062 | 72 | 6.4 |
| MOTHE_c13760 | spore coat associated protein JA (CotJA) | 322 | 4 | 6.2 |
| MOTHE_c07510 | small acid-soluble spore protein alpha/beta type | 430 | 8 | 5.8 |
| MOTHE_c18540 | L-lactate dehydrogenase | 3.139 | 73 | 5.4 |
| MOTHE_c03870 | Electron transfer subunit | 5.440 | 29 | 5.3 |
| MOTHE_c13970 | ferrous iron transport protein A | 35 | 1 | 5.3 |
| MOTHE_c03880 | Cytochrome b_556_-containing membrane subunit | 1.657 | 44 | 5.2 |
| MOTHE_c03860 | Formate dehydrogenase FdhA2 | 4.654 | 124 | 5.2 |
| MOTHE_c09110 | RNA polymerase sigma factor | 1.282 | 34 | 5.2 |
| MOTHE_c13770 | CotJB protein | 391 | 10 | 5.2 |
| MOTHE_c00600 | sporulation-specific protease YabG | 517 | 15 | 5.0 |
| MOTHE_c09170 | putative ABC transporter ATP-binding protein YxlF | 40.403 | 1.188 | 5.0 |
| MOTHE_c09730 | putative manganese catalase | 2.876 | 90 | 5.0 |
| MOTHE_c09180 | ABC-2 family transporter protein | 24.576 | 765 | 4.9 |
| MOTHE_c09150 | putative ECF RNA polymerase sigma factor SigI | 17.548 | 570 | 4.9 |
| MOTHE_c00670 | putative sporulation-specific glycosylase YdhD | 245 | 8 | 4.8 |
| MOTHE_c13780 | putative manganese catalase | 2.458 | 86 | 4.8 |
| MOTHE_c14830 | dodecin | 834 | 30 | 4.7 |
| MOTHE_c16670 | glycosyl transferase family 2 | 498 | 19 | 4.7 |
| MOTHE_c13750 | chaperone DmsD | 454 | 18 | 4.6 |
| MOTHE_c13740 | DMSO reductase subunit DmsA3 | 3.782 | 162 | 4.5 |
| MOTHE_c00630 | electron transfer flavoprotein-ubiquinone oxidoreductase EtfA | 3.826 | 188 | 4.3 |
| MOTHE_c16390 | NAD(P)H-quinone oxidoreductase subunit I. chloroplastic | 13.082 | 679 | 4.3 |
| MOTHE_c07520 | heat induced stress protein YflT | 2.051 | 102 | 4.3 |
| MOTHE_c16680 | N,N'-diacetylbacillosaminyl-diphospho-undecaprenol alpha-1,3-N-acetylgalactosaminyltransferase | 15.676 | 38 | 4.2 |
| MOTHE_c00620 | electron transfer flavoprotein-ubiquinone oxidoreductase EtfB | 4.059 | 212 | 4.2 |
| MOTHE_c13730 | DMSO reductase subunit DmsB3 | 551 | 29 | 4.2 |
| MOTHE_c00640 | electron transfer flavoprotein-ubiquinone oxidoreductase EtfC | 3.824 | 205 | 4.2 |
| MOTHE_c23710 | putative oxidoreductase YdhV | 3.397 | 183 | 4.2 |
| MOTHE_c00660 | bacterioferritin | 376 | 21 | 4.1 |
| MOTHE_c00370 | NADH-dependent phenylglyoxylate dehydrogenase subunit alpha | 5.864 | 137 | 4.1 |
| MOTHE_c15840 | NTE family protein RssA | 1.558 | 91 | 4.1 |
| MOTHE_c17950 | serine dehydrogenase proteinase | 402 | 24 | 4.0 |
| MOTHE_c14410 | succinate dehydrogenase/fumarate reductase iron-sulfur subunit | 2.858 | 176 | 4.0 |
| MOTHE_c01220 | bacillibactin exporter | 861 | 52 | 4.0 |
| MOTHE_c13720 | SAM radical protein PpqE2 | 15.286 | 100 | 4.0 |
| MOTHE_c10310 | translocation-enhancing protein TepA | 628 | 40 | 3.9 |
| MOTHE_c20730 | subtilisin E precursor | 1.366 | 89 | 3.9 |
| MOTHE_c19080 | small acid-soluble spore protein alpha/beta type | 14 | 1 | 3.9 |
| MOTHE_c00380 | 2-oxoglutarate oxidoreductase subunit KorB | 1.102 | 73 | 3.9 |
| MOTHE_c14430 | nitric oxide reductase FlRd-NAD^+^ reductase | 5.446 | 370 | 3.9 |
| MOTHE_c14500 | spore germination protein B1 | 7.482 | 73 | 3.8 |
| MOTHE_c16650 | GDP-mannose-dependent alpha-(1-6)-phosphatidylinositol monomannoside mannosyltransferase | 1.266 | 87 | 3.8 |
| MOTHE_c00390 | pyruvate synthase subunit PorC | 491 | 34 | 3.8 |
| MOTHE_c16380 | sulfite reductase. dissimilatory-type subunit gamma | 17.427 | 1.234 | 3.8 |
| MOTHE_c13960 | ferrous iron transport protein B | 313 | 22 | 3.8 |
| MOTHE_c09710 | spore germination protein B1 | 145 | 10 | 3.8 |
| MOTHE_c14420 | heterodisulfide reductase subunit A2 | 20.018 | 939 | 3.7 |
| MOTHE_c00650 | ferredoxin-like protein FixX | 45 | 3 | 3.7 |
| MOTHE_c02120 | sporulation protein YpeB | 1.849 | 139 | 3.7 |
| MOTHE_c19740 | galactose-1-phosphate uridylyltransferase | 967 | 73 | 3.7 |
| MOTHE_c00400 | NAD(P)H-quinone oxidoreductase subunit I. chloroplastic | 90 | 6 | 3.7 |
| MOTHE_c25900 | spore protein SP21 | 650 | 50 | 3.6 |
| MOTHE_c19530 | 1,4-alpha-glucan branching enzyme | 993 | 80 | 3.6 |
| MOTHE_c19750 | glycogen synthase | 1.354 | 109 | 3.6 |
| MOTHE_c02130 | spore cortex-lytic enzyme precursor | 483 | 39 | 3.6 |
| MOTHE_c17960 | coat F domain protein | 11.879 | 168 | 3.5 |
| MOTHE_c01050 | putative L.D-transpeptidase YkuD | 395 | 34 | 3.5 |
| MOTHE_c06770 | putative amino acid permease YhdG | 1.038 | 94 | 3.4 |
| MOTHE_c19730 | glycogen synthase | 1.362 | 124 | 3.4 |
| MOTHE_c13700 | DMSO reductase subunit DmsB2 | 1.206 | 113 | 3.4 |
| MOTHE_c13950 | ferrous iron transport protein B | 433 | 40 | 3.4 |
| MOTHE_c13710 | DMSO reductase subunit DmsA2 | 8.901 | 624 | 3.3 |
| MOTHE_c22290 | acetyl-coenzyme A synthetase | 29.829 | 2.077 | 3.3 |
| MOTHE_c23730 | glucose-6-phosphate 1-dehydrogenase | 382 | 38 | 3.3 |
| MOTHE_c04100 | calcium-transporting ATPase | 16.183 | 164 | 3.2 |
| MOTHE_c20600 | spore coat protein F precursor | 18.388 | 393 | 3.2 |
| MOTHE_c13690 | DMSO reductase anchor subunit (DmsC) | 1.547 | 164 | 3.2 |
| MOTHE_c16370 | sulfurtransferase TusE | 1.946 | 214 | 3.2 |
| MOTHE_c17740 | YtxC-like family protein | 5.109 | 103 | 3.2 |
| MOTHE_c18310 | malto-oligosyltrehalose trehalohydrolase | 6.926 | 165 | 3.1 |
| MOTHE_c15780 | RNA polymerase sigma-28 factor precursor | 666 | 75 | 3.1 |
| MOTHE_c09690 | spore germination protein B3 precursor | 124 | 14 | 3.1 |
| MOTHE_c01240 | phosphosulfolactate synthase | 2.776 | 322 | 3.1 |
| MOTHE_c16550 | PRC-barrel domain protein | 2.197 | 259 | 3.1 |
| MOTHE_c16350 | sulfite reductase. dissimilatory-type subunit beta DsrB2 | 11.854 | 6.884 | 3.1 |
| MOTHE_c13110 | stage II sporulation protein P (SpoIIP) | 12.472 | 335 | 3.0 |
| MOTHE_c16360 | sulfite reductase. dissimilatory-type subunit alpha | 24.035 | 7.343 | 3.0 |
| MOTHE_c15990 | sulfite reductase. dissimilatory-type subunit beta DsrB1 | 30.201 | 3.744 | 3.0 |
| MOTHE_c23670 | germination-specific N-acetylmuramoyl-L-alanine amidase precursor | 19.884 | 533 | 3.0 |
| MOTHE_c16340 | cobyrinic acid A,C-diamide synthase | 16.643 | 1.138 | 3.0 |
| MOTHE_c14510 | spore germination protein B3 precursor | 327 | 40 | 3.0 |
| MOTHE_c20220 | putative sulfoacetate--CoA ligase | 312 | 39 | 2.9 |
| MOTHE_c18320 | glycosyl hydrolase family 57 | 1.906 | 242 | 2.9 |
| MOTHE_c21630 | branched-chain-amino-acid aminotransferase | 17.329 | 413 | 2.9 |
| MOTHE_c15980 | dissimilatory sulfite reductase D (DsrD) | 848 | 112 | 2.9 |
| MOTHE_c18330 | maltooligosyl trehalose synthase | 1.630 | 216 | 2.9 |
| MOTHE_c16000 | sulfite reductase. dissimilatory-type subunit alpha | 34.809 | 12.273 | 2.9 |
| MOTHE_c23720 | kynurenine formamidase | 182 | 24 | 2.9 |
| MOTHE_c08000 | stage II sporulation protein R | 1.264 | 174 | 2.8 |
| MOTHE_c20270 | 2-oxoglutarate oxidoreductase subunit KorB | 216 | 30 | 2.8 |
| MOTHE_c20290 | phenylalanyl-tRNA synthetase subunit beta | 53 | 7 | 2.8 |
| MOTHE_c21620 | aminodeoxychorismate synthase component 1 | 8.275 | 1.011 | 2.7 |
| MOTHE_c06510 | putative murein peptide carboxypeptidase | 338 | 49 | 2.7 |
| MOTHE_c18340 | trehalose synthase | 710 | 103 | 2.7 |
| MOTHE_c12460 | D-aminopeptidase | 1.084 | 161 | 2.7 |
| MOTHE_c17260 | electron bifurcating hydrogenase subunit HydA | 3.451 | 500 | 2.7 |
| MOTHE_c16010 | nitrate reductase gamma subunit | 4.507 | 691 | 2.7 |
| MOTHE_c07990 | RNA polymerase sigma-F factor | 1.455 | 225 | 2.7 |
| MOTHE_c20240 | putative aliphatic sulfonates transport permease protein SsuC | 109 | 16 | 2.6 |
| MOTHE_c17280 | electron bifurcating hydrogenase subunit HydC | 442 | 67 | 2.6 |
| MOTHE_c22300 | sensor histidine kinase YehU | 978 | 155 | 2.6 |
| MOTHE_c20230 | putative aliphatic sulfonates-binding protein precursor | 165 | 26 | 2.6 |
| MOTHE_c24970 | serine protease Do-like HtrB | 974 | 161 | 2.6 |
| MOTHE_c08790 | germination protease precursor | 1.466 | 240 | 2.5 |
| MOTHE_c17270 | electron bifurcating hydrogenase subunit HydB | 3.154 | 522 | 2.5 |
| MOTHE_c16020 | polysulfide reductase. NrfD | 6.115 | 1.048 | 2.5 |
| MOTHE_c12080 | DNA polymerase IV | 1.164 | 189 | 2.5 |
| MOTHE_c16530 | AI-2 transport protein TqsA | 1.451 | 247 | 2.5 |
| MOTHE_c15970 | beta-barrel assembly-enhancing protease | 827 | 150 | 2.5 |
| MOTHE_c09680 | spore germination protein YndE | 68 | 11 | 2.5 |
| MOTHE_c16030 | tetrathionate reductase subunit B precursor | 4.029 | 730 | 2.5 |
| MOTHE_c01700 | heptaprenyl diphosphate synthase component 2 | 633 | 113 | 2.4 |
| MOTHE_c12170 | Low-affinity inorganic phosphate transporter 1 | 441 | 79 | 2.4 |
| MOTHE_c19050 | sporulation membrane protein YtrH | 314 | 56 | 2.4 |
| MOTHE_c00920 | spore protein YabP | 1.146 | 211 | 2.4 |
| MOTHE_c10790 | stage V sporulation protein K | 618 | 112 | 2.4 |
| MOTHE_c00930 | spore cortex protein YabQ | 2.101 | 389 | 2.4 |
| MOTHE_c14490 | spore germination protein YndE | 140 | 26 | 2.4 |
| MOTHE_c20260 | 2-oxoglutarate oxidoreductase subunit KorA | 203 | 38 | 2.3 |
| MOTHE_c07900 | UDP-N-acetylmuramate--L-alanine ligase | 5.185 | 1.005 | 2.3 |
| MOTHE_c20280 | 2-oxoglutarate ferredoxin oxidoreductase subunit gamma | 95 | 18 | 2.3 |
| MOTHE_c09480 | N-acetylmuramoyl-L-alanine amidase LytC precursor | 3.209 | 631 | 2.3 |
| MOTHE_c08820 | thioredoxin | 24.673 | 909 | 2.3 |
| MOTHE_c20210 | aliphatic sulfonates import ATP-binding protein SsuB | 105 | 20 | 2.3 |
| MOTHE_c22310 | sensory transduction protein LytR | 378 | 77 | 2.3 |
| MOTHE_c20880 | spore germination protein B3 precursor | 531 | 106 | 2.3 |
| MOTHE_c12160 | putative pit accessory protein | 299 | 61 | 2.3 |
| MOTHE_c13580 | spore germination protein B1 | 1.293 | 262 | 2.3 |
| MOTHE_c21260 | lipid II flippase FtsW | 41 | 8 | 2.2 |
| MOTHE_c06790 | peptidoglycan-N-acetylglucosamine deacetylase | 21.930 | 24.089 | 2.2 |
| MOTHE_c13610 | SpoVA protein | 548 | 112 | 2.2 |
| MOTHE_c06330 | vancomycin B-type resistance protein VanW | 12.584 | 1.449 | 2.2 |
| MOTHE_c07920 | UDP-N-acetylglucosamine 1-carboxyvinyltransferase 1 | 3.306 | 702 | 2.2 |
| MOTHE_c18580 | thiol:disulfide interchange protein DsbD | 232 | 50 | 2.2 |
| MOTHE_c16050 | succinate dehydrogenase/fumarate reductase iron-sulfur subunit | 14.400 | 3.247 | 2.1 |
| MOTHE_c25000 | thioredoxin reductase | 3.755 | 851 | 2.1 |
| MOTHE_c09490 | F420-0:gamma-glutamyl ligase | 1.315 | 290 | 2.1 |
| MOTHE_c00580 | spore cortex-lytic enzyme precursor | 76 | 17 | 2.1 |
| MOTHE_c12820 | asparagine synthetase [glutamine-hydrolyzing] 3 | 6.087 | 1.410 | 2.1 |
| MOTHE_c13600 | stage V sporulation protein AD | 9.441 | 289 | 2.1 |
| MOTHE_c08120 | general stress protein 16O | 323 | 75 | 2.0 |
| MOTHE_c16330 | HTH-type transcriptional regulator SinR | 16.801 | 587 | 2.0 |
| MOTHE_c00870 | stage V sporulation protein T | 5.946 | 1.402 | 2.0 |
| MOTHE_c13560 | 2-hydroxyglutaryl-CoA dehydratase. D-component | 1.169 | 281 | 2.0 |
| MOTHE_c04470 | epoxyqueuosine reductase | 4.982 | 1.211 | 2.0 |
| MOTHE_c11080 | stage V sporulation protein B | 10.042 | 451 | 2.0 |
| MOTHE_c04090 | Hsp20/alpha crystallin family protein | 228 | 55 | 2.0 |
| MOTHE_c00440 | peptidoglycan-N-acetylmuramic acid deacetylase PdaA precursor | 15.794 | 588 | 2.0 |

**Supplementary table 4: The most downregulated genes of *M. thermoacetica* during growth on methanol + DMSO.**

| **gene** | **annotation** | **substrate** | | **Log_2_ (fold change)** |
| --- | --- | --- | --- | --- |
|  |  | **methanol + DMSO** | **methanol** |  |
| MOTHE_c14240 | ferrous iron transport protein A | 1 | 12 | -3.0 |
| MOTHE_c21690 | Methyltransferases MtvA | 1.520 | 9.522 | -2.6 |
| MOTHE_c11800 | septum site-determining protein MinD | 1.350 | 8.306 | -2.6 |
| MOTHE_c14220 | ferrous iron transport protein B | 77 | 526 | -2.6 |
| MOTHE_c21700 | corrinoid protein MtvC4 | 1.009 | 6.204 | -2.6 |
| MOTHE_c11730 | Methyltransferase (WLP) | 6.059 | 35.627 | -2.6 |
| MOTHE_c11740 | corrinoid/iron-sulfur protein small subunit CoFeSP (WLP) | 11.592 | 41.782 | -2.5 |
| MOTHE_c11750 | maturation protein AcsF | 3.775 | 21.668 | -2.5 |
| MOTHE_c11760 | putative corrinoid activating enzyme | 18.099 | 104.160 | -2.5 |
| MOTHE_c11770 | corrinoid/iron-sulfur protein large subunit CoFeSP (WLP) | 22.182 | 59.214 | -2.5 |
| MOTHE_c14210 | cyclic di-GMP phosphodiesterase response regulator RpfG | 44 | 307 | -2.5 |
| MOTHE_c01150 | formyl-THF synthetase | 9.396 | 52.957 | -2.5 |
| MOTHE_c11780 | carbon monoxide dehydrogenase/acetyl-CoA synthase subunit alpha | 12.597 | 68.282 | -2.4 |
| MOTHE_c11790 | carbon monoxide dehydrogenase/acetyl-CoA synthase subunit beta | 19.446 | 60.407 | -2.4 |
| MOTHE_c17220 | transcriptional regulatory protein ZraR | 336 | 10.578 | -2.4 |
| MOTHE_c23810 | lactate utilization protein C | 104 | 560 | -2.4 |
| MOTHE_c23790 | HTH-type transcriptional regulator LutR | 130 | 669 | -2.3 |
| MOTHE_c11720 | MTHFR subunit HdrC | 1.975 | 9.208 | -2.2 |
| MOTHE_c23820 | anaerobic glycerol-3-phosphate dehydrogenase subunit C | 281 | 1.371 | -2.2 |
| MOTHE_c20980 | phosphoribosylformylglycinamidine synthase subunit PurS | 69 | 317 | -2.2 |
| MOTHE_c11710 | cysteine-rich domain protein | 18.092 | 28.371 | -2.1 |
| MOTHE_c17290 | multifunctional cyclase-dehydratase-3-O-methyl transferase TcmN | 563 | 2.566 | -2.1 |
| MOTHE_c23800 | lactate utilization protein B | 529 | 9.529 | -2.1 |
| MOTHE_c17300 | nicotinate-nucleotide--dimethylbenzimidazole phosphoribosyltransferase | 811 | 3.553 | -2.1 |
| MOTHE_c21680 | sensor histidine kinase YpdA | 356 | 1.535 | -2.1 |
| MOTHE_c14230 | FeoA domain protein | 4 | 21 | -2.0 |
| MOTHE_c17310 | phosphomethylpyrimidine synthase | 947 | 16.496 | -2.0 |
| MOTHE_c23830 | putative FAD-linked oxidoreductase | 515 | 16.765 | -2.0 |
| MOTHE_c15680 | primary amine oxidase precursor | 0 | 2 | -2.0 |
